# Development of a Model for Predicting Chronological Age Based on DNA Methylation Data from Buccal Swabs Using Ion Torrent S5 Technology

**DOI:** 10.3390/ijms27157062

**Published:** 2026-08-06

**Authors:** Yecith D. Puerto-Parra, Antonio Gómez-Martín, Francisco Andújar-Vera, María Saiz, Diana C. Vinueza-Espinosa, Olga López-Guarnido, Juan Carlos Álvarez, José Antonio Lorente

**Affiliations:** 1 Laboratory of Genetic Identification (LABIGEN-UGR), Department of Legal Medicine, Faculty of Medicine, University of Granada, 18016 Granada, Spain; yecithpuerto@correo.ugr.es (Y.D.P.-P.); msaiz@ugr.es (M.S.); dvinuezaespinosa@ugr.es (D.C.V.-E.); jlorente@ugr.es (J.A.L.); 2 Bioinformatics Unit, Instituto de Investigación Biosanitaria ibs.GRANADA, 18012 Granada, Spain; antonio.gm.gr@gmail.com (A.G.-M.); fandujar@fibao.es (F.A.-V.); 3 Department of Legal Medicine, Toxicology and Physical Anthropology, Faculty of Medicine, University of Granada, 18016 Granada, Spain; olga@ugr.es

**Keywords:** DNA methylation, forensic age estimation, CpG islands, epigenetics, buccal swab, bisulfite conversion, Elastic Net

## Abstract

Age estimation using DNA methylation analysis is one of the tools currently used in forensic science to facilitate human identification. A total of 117 buccal swab samples from individuals aged 18 to 67 years were analyzed using a custom panel encompassing 610 CpG sites across 12 age-related genes. The analysis was performed using the amplicon-based technology Ion AmpliSeq™, which incorporates Ion Torrent S5 sequencing (Thermo Fisher Scientific, Waltham, MA, USA). The resulting methylation data were used to develop and compare age-prediction models based on Watson-strand, Crick-strand, and combined Watson + Crick methylation matrices. After candidate CpG selection and model reduction, the final Watson-strand model used 5 CpG sites from the age-related genes C1orf132, ELOVL2, KLF14, PDE4C and TRIM59 and was fitted using Elastic Net regression with alpha grid search, lambda selection and internal cross-validation. Model validation yielded prediction results with a mean absolute error of 4.42 years for the Watson matrix, 4.75 years for the Crick matrix, and 5.08 years for the WC matrix. These results allow for the evaluation of the suitability of using Ion AmpliSeq™ technology in forensic age estimation studies based on DNA methylation analysis.

## 1. Introduction

Standard forensic DNA methods rely primarily on comparative analysis, which aims to match DNA profiles from samples found at crime scenes with those of known suspects, such as samples stored in forensic DNA databases. Consequently, individuals whose DNA profiles are unknown to investigators cannot be easily identified [1,2,3,4].

The predictive power of DNA information is increasingly used in forensic medicine, leading to the establishment of forensic DNA phenotyping (FDP). FDP involves predicting an individual’s external visible characteristics (EVCs), such as appearance, biogeographical ancestry, and age, from DNA extracted from human biological samples found at crime scenes [1,5,6].

The FDP is particularly useful in criminal cases where no match is identified in forensic short tandem repeat (STR) profiles, where the donor’s identity remains unknown to investigating authorities and, therefore, their STR profile is unavailable for comparison [7,8]. FDP tools consist of two key components: (i) a forensically validated multiplex genotyping tool that can analyze all relevant predictive DNA markers in crime scene samples, using suitable DNA technology that accommodates low-quality and low-quantity DNA, and (ii) a mathematical predictive model to obtain appearance and ancestry probabilities, and to estimate age from genetic and epigenetic data, respectively, obtained using the multiplex genotyping tool [7].

Until recently, pyrosequencing, minisequencing, and mass spectrometry were the most common methods for DNA methylation analysis [9,10,11]. However, increasingly, massively parallel sequencing (MPS) protocols for SNP analysis are being adapted for this purpose, although this shift has brought several challenges, such as the use of sodium bisulfite conversion, difficulties in primer design due to the reduced complexity of the DNA sequence after conversion, and post-conversion DNA degradation. New technologies are expanding the potential applications of DNA methylation analysis in forensic genetics, potentially enabling the analysis of a wider range of markers, thereby increasing the accuracy of age prediction and integrating diverse applications onto a single multiplex platform, such as using DNA methylation analysis to predict age, identify tissue types, and profile behaviors. Age estimation using DNA methylation analysis is sufficiently accurate for practical forensic applications [1,7,12,13,14,15].

The aging process is associated with epigenetic modifications, including DNA methylation at specific CpG sites in the human genome [16,17]. At certain CpG sites, methylation levels correlate almost linearly with age, making them suitable biomarkers for age prediction in forensic applications [4,7]. While the epigenetic clock concept was introduced in 2011 [18], prediction models have been continuously improved through the analysis of an increasingly broad and refined list of methylation markers, as well as the integration of various clinical biomarkers [19,20]. Age estimation can be particularly useful in forensic investigations to complement the prediction of visible external features and the inference of biogeographical ancestry, thereby providing a more complete picture of an unknown individual [8,21,22,23,24]. In forensic science, age prediction can be beneficial for: (i) narrowing the pool of potential suspects; (ii) facilitating the prediction of certain visible external features correlated with age [3,25,26]; (iii) improving human identification from skeletal remains; (iv) determining the applicability of legal provisions that depend on the age of the individual in question; and (v) playing a fundamental role in immigration cases where the identity and age of individuals are uncertain [3,9,27,28].

Most current forensic studies on age-correlated methylation patterns and model validation rely on blood samples, with fewer models available for saliva and buccal swabs. However, the accuracy of estimates for saliva and buccal swabs is comparable to that of blood samples, with mean absolute errors (MAE) ranging from 3 to 6 years [29,30,31,32,33,34,35].

Given the diversity of NGS platforms used in forensic laboratories (e.g., Illumina, Ion Torrent S5, Oxford Nanopore, PacBio), technology transfer could be crucial for the widespread implementation of these tools. The difference between these platforms lies in the size of the reading target (short or long) and the accuracy of base calling.

The development of age prediction models is based on the application of computational algorithms capable of generating predictive models through the intelligent autonomous analysis of relatively large and often unstructured data, which is known as Machine Learning [36].

Current applications of machine learning in forensic biology can be arbitrarily divided into: (i) applications related to human identification, such as the efficient designation of STR alleles from data generated by capillary electrophoresis (CE) or NGS, while filtering out potential artifacts and assisting with the interpretation of DNA mixtures, such as estimating the number of contributors (NoC) to a DNA mixture; (ii) applications related to forensic intelligence, such as forensic molecular phenotyping and biogeographical ancestry prediction; (iii) applications related to increasing the probative value of DNA evidence, such as forensic microbiology (e.g., estimating the postmortem interval), confirming the source of biological tissue, and reporting activity levels. Other potential applications of ML (Machine Learning) methods in the field of forensic DNA include forensic intelligence and inference, such as predicting the origin of bodily fluids [37,38], the features of visual appearance, biogeographical ancestry, biological age [1,39,40] and the estimation of the post-mortem interval.

DNA methylation-based age estimators are typically constructed using a transformed regression of chronological age on a set of CpG sites, employing a supervised machine learning method. Each of these methods is designed with mathematical algorithms that incorporate the DNA methylation levels detected by the CpG site set and, through the selection of specific age-related variables, allow for the development of an age prediction model [41].

The method of choice should be determined by the (non)normal distribution of CpG methylation, (non)linear relationships, collinearity, non-constant variance, or heteroscedasticity in our dataset [42], indicating the importance of optimal and appropriate selection of DNA methylation markers. When using these DNA methylation-based age estimation models, it is important to consider that prediction errors often increase in an age-dependent manner, and the resulting accuracies decrease for older individuals [43].

Initial approaches to predicting age based on DNA methylation focused on the use of linear regression. However, the challenge posed by the relatively small magnitude of methylation variation with age has led current research to question the suitability of linear models for this type of analysis and to investigate new options [44].

Among the methods used to develop age prediction models, simple and multiple linear regression methods stand out [36,45]: ordinary least squares regression [43,46]; weighted least squares [36,45,46]; multivariate quantile regression [47,48]; support vector regression [1,36,44]; random forest regression [45]; gradient boosting regression [49].

In the present study, methylation data generated from a custom Ion AmpliSeq™/Ion Torrent S5 panel were used to develop and compare age-prediction models based on Watson-strand, Crick-strand, and combined Watson + Crick methylation matrices. After candidate CpG screening and model reduction, the final Watson-strand model retained 5 CpGs in five age-related genes (C1orf132, ELOVL2, KLF14, PDE4C, and TRIM59) and was fitted using Elastic Net regression with alpha grid search and lambda selection under internal cross-validation. The novelty of this work is threefold: (1) to our knowledge it is the first buccal-swab epigenetic age model developed natively on Ion AmpliSeq/Ion Torrent S5, avoiding cross-platform corrections; (2) it uses a broad custom panel (12 age-related genes, 610 CpGs, 135 amplicons) integrating loci usually studied in isolation; and (3) it evaluates Watson and Crick strands separately—rarely done—showing that strand orientation affects predictive signal. This study evaluates the suitability of Ion AmpliSeq™ technology for forensic age estimation based on DNA methylation analysis.

The Results section first summarizes the custom panel design, sequencing-quality parameters, sample selection, model development, and model performance. Detailed methodological information on sample processing, bisulfite conversion, sequencing, generation of the Watson, Crick and Watson + Crick matrices, cross-validation schemes, algorithm comparison, and model-selection procedures is provided in Section 4.

## 2. Results

### 2.1. Design of a Custom Methylation Panel

Of the 649 CpG sites selected, 39 were not covered due to their location in repetitive or otherwise technically challenging genomic regions, resulting in a 94% success rate for the customized methylation panel. Ultimately, 610 CpG sites across 12 age-related genes of interest were analyzed within 135 amplicons, distributed between 2 pools of 67 and 68 amplicons, respectively, including primers for 2 lambda control amplicons. For 74% of the CpG sites (451 of 610), primers that targeted both strands were successfully designed. The remaining 26% (159 of 610 CpG sites) were analyzed using primers that were specific to either the Watson or Crick strand. The number of amplicons that were assigned to each CpG site varied from 1 to 4, depending on whether CpG sites were present within the primer binding sites.

It is important to mention that, considering that during the search and identification of CpG sites in genomic regions between 180 and 900 bp near the promoter regions of each gene, 649 CpG sites were identified, which were the target for detection in the custom DNA methylation panel. Thus, during the analysis of the sequencing results obtained from each sample, an additional 64 CpG sites were detected, bringing the total number of CpG sites targeted for detection by the custom panel to 676. This is due to the panel’s amplicon design, which allowed for the amplification of CpG sites near the regions of interest.

Supplementary Material S1 contains relevant information regarding the custom DNA methylation panel in this study, such as the CpG sites that were included and a complete list of the 135 amplicons that were targeted by the panel. Further, Supplementary Material S2 comprises a list of the 71 CpG sites that were identified as being essential for verifying the reproducibility using Ion AmpliSeq technology in this study.

### 2.2. Read Depth Analysis

All sequencing runs achieved high total coverage. A total of 12 to 16 barcoded libraries were co-sequenced per run, optimizing the chip’s capacity.

The average total reads that were obtained per chip was 6.08 ± 1.31 M (median: 6.09 M; range: 3.41–7.83 M), consistent with the expected output range for the Ion 530™ chip (typically 6–9 M reads), as reported by the manufacturer. The average percentage of usable reads per chip was 32.33% ± 6.41%, also falling within the manufacturer’s values (>30%). The average chip loading percentage was 51.50 ± 8.42%, which is also within the range that has been reported by the manufacturer (40–60%). The average percentage of polyclonality per chip was 28.08% ± 3.34%, slightly exceeding the acceptable range as specified by the manufacturer (20–25%), which may be related to slight variations in the final concentration of the library pool during the annealing process, despite all libraries having a concentration of 50 pM. However, this parameter can be adjusted in the run template by modifying the polyclonality filter settings. The average enrichment percentage per chip was 97.42% ± 2.54%, meeting the manufacturer’s recommended minimum of 95%.

The average number of reads that covered the target CpGs was 181.6 ± 60.5 k (median: 177.7 k; range: 87.5–280 k). The mapping efficiency was high (84.67 ± 5%). On average, 64.77% ± 13.75% of reads mapped to the target (fraction of mapped reads that fall in the target regions), with an average uniformity (fraction of target bases with coverage ≥ 0.2× the average coverage, evenness) of 69.19% ± 9.28%.

The average number of mapped reads that covered the target CpGs on the Watson strand was 126.1 ± 33 k (median: 106.7 k; range: 72.6–171.2 k), compared with 114.9 ± 29.4 k (median: 127.4 k; range: 74.1–170.3 k) on the Crick strand.

### 2.3. Sample Selection for Prediction Model Design

As a selection criterion for the age prediction model, the minimum threshold of 50 CpG reads (by summing methylated and nonmethylated reads in each amplicon) was verified at the 71 CpG sites that were identified as being essential for verifying reproducibility with Ion AmpliSeq technology.

Thus, 103 samples (67.77%) reached the minimum-reads threshold at the 71 essential CpG sites, which were covered by a predetermined number of amplicons, according to the final panel design. Based on this selection criterion, 49 samples (32.23%) did not reach the minimum threshold of 50 reads for some of the 71 essential CpG sites. Of these samples, 16 were selected, taking into account samples from age groups in which at least 2 samples per year of age were required, and the library was reamplified. Results that exceeded the minimum read threshold at the 71 essential CpG sites were obtained in 7 of these samples. Finally, 7 additional samples that met the reading threshold (50 reads) were selected for some of the 71 essential CpG sites, even though the threshold was not met at all sites. Therefore, the total number of samples selected for the age prediction model was 117.

### 2.4. Overview of Age Prediction Model Development

#### 2.4.1. Quality Control and Final Dataset

Of the initial 152 samples, 117 passed the 50-read coverage threshold at all 71 essential CpG sites. After subsequent filtering (coverage, missing data, low variance, and outlier exclusion), the final dataset comprised 111 (Watson), 109 (Crick), and 110 (Watson + Crick) samples (Table 1).

#### 2.4.2. Univariate Screening Results

Univariate screening identified CpGs with strong age associations (|ρ| ≥ 0.50, IQR ≥ 5, R^2^ ≥ 0.25) across all three matrices. After collinearity reduction (|r| > 0.85), the candidate sets comprised approximately 19 (Watson), 18 (Crick), and 16 (Watson + Crick) CpGs spanning the 12 target genes.

#### 2.4.3. Algorithm-Comparison Results

A full comparison of the six algorithms under identical 10-fold × 5-repeat CV is provided in Supplementary Material S3 (Table S7). Elastic Net achieved the lowest MAE for the Watson matrix (3.94 ± 0.13 years), essentially tied with LASSO and SVM (3.96 ± 0.13 years). For Crick and WC, tree- and kernel-based methods were marginally better, but the differences between the best-performing algorithms generally overlapped within approximately one standard error. Elastic Net was selected for the final Watson-strand model because it combines competitive accuracy in the selected matrix with coefficient-level interpretability, stability under multicollinearity and parsimony—properties that are decisive for a forensic, reportable model.

#### 2.4.4. Representative CpG Panel

The representative selection procedure reduced the model to 5 (Watson), 6 (Crick), and 7 (Watson + Crick) CpGs, with natural splines applied selectively to CpGs showing significant non-linearity (ΔAIC > 10, adjusted *p* < 0.10) (Supplementary Material S3, Table S1).

#### 2.4.5. Nested Cross-Validation

Nested CV confirmed the generalizability of the reduced panels, with Watson achieving MAE = 4.70 years, RMSE (root mean square error) = 5.59 years, and R^2^ = 0.87, superior to Crick (MAE = 5.30 years) and Watson + Crick (MAE = 5.73 years) (Supplementary Material S3, Table S2).

#### 2.4.6. CpG Selection Stability

Core CpGs (e.g., C1orf132, KLF14, TRIM59) appeared in 100% of outer folds, confirming robust selection. Peripheral CpGs with frequency < 60% were excluded from the final panel (Supplementary Material S3, Table S3).

#### 2.4.7. Final Model Performance (CV 10 × 5)

The expected out-of-sample performance was MAE = 4.70 years (nested CV). In repeated internal validation, an MAE of 4.42 years (SE = 0.12; RMSE = 5.27 years, R^2^ = 0.89) was obtained for the final Watson-strand model. The fit over the full training set is shown for descriptive purposes only, using 5 CpGs with 2 natural splines (Supplementary Material S3, Table S4).

#### 2.4.8. Training Calibration

All three models showed negligible mean bias (≈0), with Watson showing the lowest SD of errors (4.81) and highest age-prediction correlation (r = 0.945) (Supplementary Material S3, Table S5).

#### 2.4.9. Model Coefficients

Table S6 and Figure S1 in Supplementary Material S3 show the largest coefficients obtained from the final Elastic Net model for the Watson strand (Supplementary Material S3, Table S6 and Figure S1).

**Figure 1:**
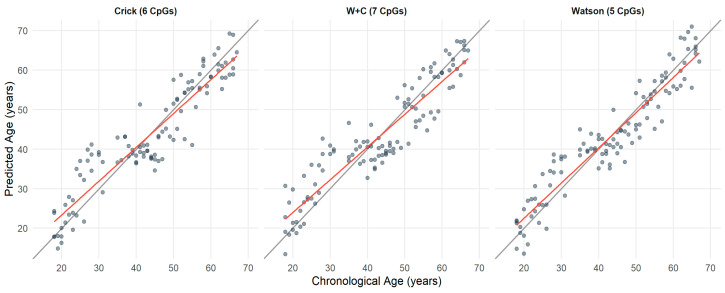
Predicted vs. chronological age for all three matrices (Watson, Crick, Watson + Crick). Each panel shows the grey identity line (perfect prediction) and the red linear regression fit.

Based on the information obtained above, the three data matrices created to develop the prediction model were compared to identify specific features in each matrix, as shown in Figure 1.

All three plots show a very clear and strong positive linear relationship. As chronological age increases, so does predicted age. This demonstrates that the selected CpG sites (whether 5, 6 or 7) are effective biomarkers for estimating age.

For the model with the Crick strand, there is a slight overestimation at younger ages (20–25 years). For the combined W + C model, which includes an additional CpG site, there is less overall dispersion, and the red line—which corresponds to the model’s actual fit—almost coincides with the grey line, which corresponds to the ‘perfect prediction’ reference line. For the model with the Watson strand, despite having the fewest CpG sites (only 5), shows an excellent fit (r = 0.945). The points appear to be clustered very tightly around the identity line, particularly in the 35 to 55-year age range.

**Figure 2:**
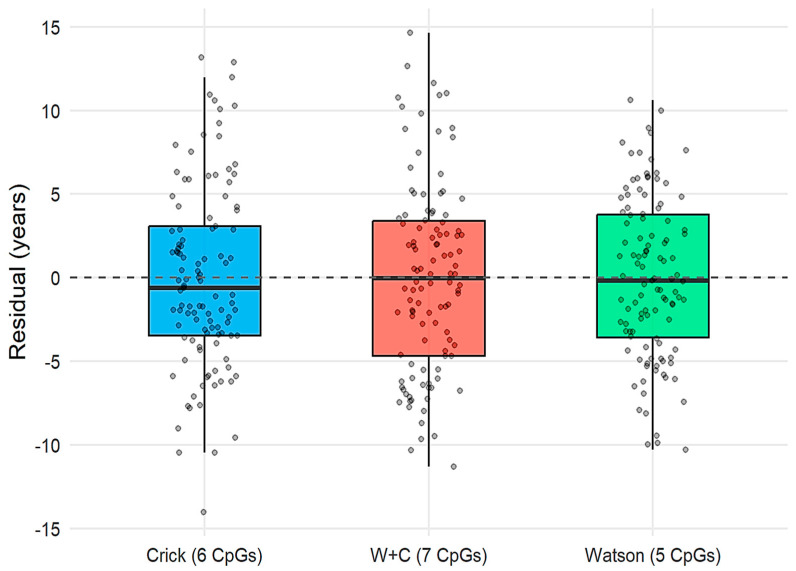
Distribution of prediction residuals by matrix. Box: IQR; whiskers: 1.5 × IQR; points: individual residuals.

During the residual analysis of the three models (see Figure 2), a relatively good fit can be observed because their medians are close to zero. For about half of the individuals (interquartile range, IQR), the prediction error is within ±4 years, while 94% fall within ±10 years; the overall cross-validated mean absolute error is 4.42 years. This indicates competitive performance, particularly given the small number of CpG sites used. As for dispersion and outliers, most of the maximum errors fall within ±10 years, with a few isolated cases of around +15 years (severe overestimation) or −14 years (severe underestimation), which is common due to individual biological variations or lifestyle factors that affect methylation [50,51,52]. Despite using different numbers of CpG sites (5, 6 or 7) and originating from different DNA strands, the distribution of the residuals is very similar across the three groups. No drastic differences are observed in the size of the boxes or in the dispersion of the points; thus, the reduction to just 5 CpGs on the Watson strand (green box) maintains robustness and a margin of error comparable to the combined W + C model of 7 CpGs (red box).

**Figure 3:**
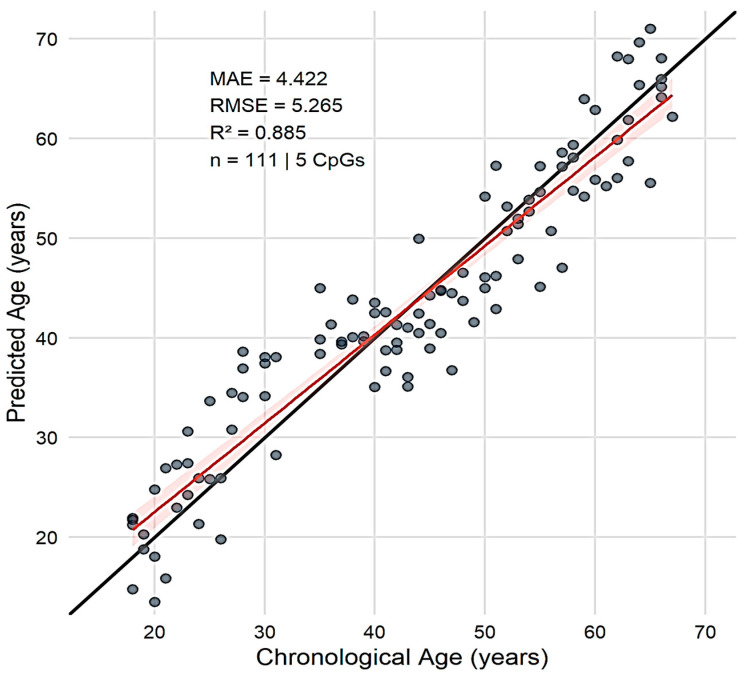
Predicted versus chronological age for the Watson-strand final model. The scatterplot illustrates the model fit for descriptive purposes, whereas the performance metrics shown (MAE = 4.422 years, RMSE = 5.265 years, R^2^ = 0.885) correspond to repeated internal cross-validation (10-fold × 5-repeat CV, where CV denotes cross-validation). The gray line represents the identity line (perfect prediction); the red line represents the linear regression fit; the shaded area indicates the 95% confidence band.

Using data from the ‘dat_W’ matrix (Watson strand), which showed the highest age-prediction correlation (r = 0.945), Figure 3 shows the relationship between chronological age and predicted age. The scatterplot is shown for descriptive purposes; the cross-validated performance metrics for the final Watson-strand model were MAE = 4.42 years, RMSE = 5.27 years, and R^2^ = 0.885, indicating good model performance with relatively low errors.

**Figure 4:**
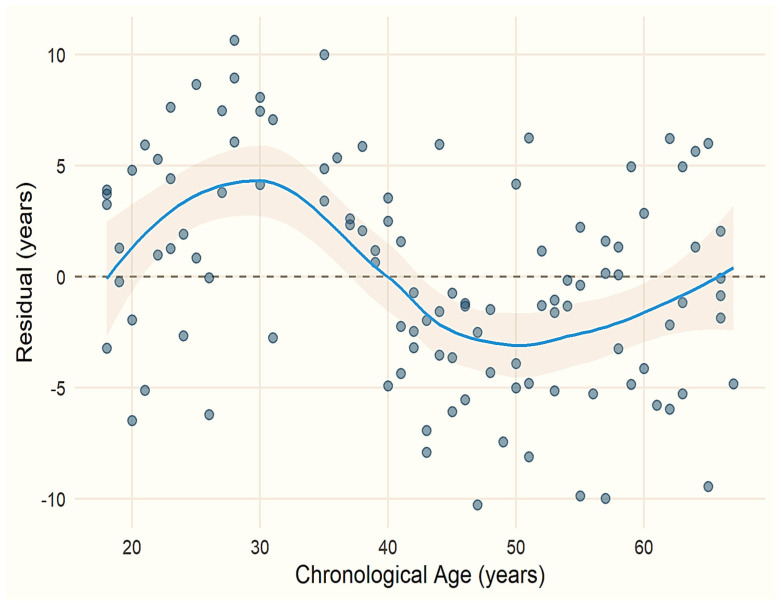
Prediction residuals (predicted − chronological age) as a function of chronological age for the final Watson-strand model fitted on the full training set (n = 111). The horizontal dashed line indicates zero bias. The blue LOESS (Locally Estimated Scatterplot Smoothing) curve is a non-parametric local-regression smoother used to reveal any age-dependent trend in the residuals without assuming a global functional form.

The model’s prediction bias was also assessed using a residual analysis. Figure 4 shows that the model exhibits systematic bias depending on age. In the 20–35 age range, the model tends to overestimate age; in the 40–55 age range, the model tends to underestimate it; and for those over 60, the bias decreases again. Most residuals remain within ±5 years, which is reasonable when compared with the mean absolute error obtained (MAE = 4.42 years).

**Figure 5:**
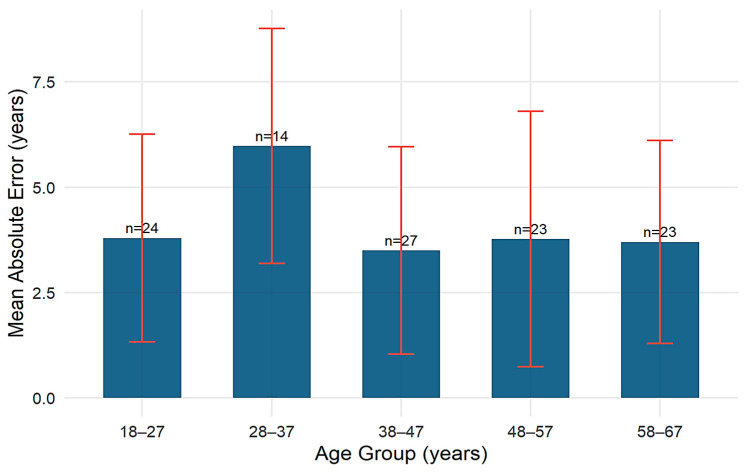
Mean absolute error (MAE) by age group for the final Watson-strand model fitted on the full training set (n = 111). Age groups were defined a priori as 10-year intervals during sampling. Error bars represent ±1 standard deviation.

When analysing the Mean Absolute Error (MAE) by age group, it can be seen that for groups A, C, D and E, the values remain relatively stable between approximately 3.5 and 4 years, with the exception of group B, where an overestimation is observed, possibly due to the sample size. The age groups were defined a priori as 10-year intervals during the sampling design (see Figure 5).

The impact of each variable on the mathematical model was assessed using the CpG sites selected for the Watson-strand model. Figure S1 in Supplementary Material S3 shows, in order of greatest to least importance, the absolute coefficients of the final model. C1orf132_CpG-5 and TRIM59_CpG-44 had coefficient magnitudes of 6.3 and 5.6, respectively, suggesting that methylation in these two genes is important in the age prediction model.

## 3. Discussion

In the current literature, many models exist for predicting epigenetic age [2,9,12,15,16,17,32,53,54]. Some of these models are designed for forensic needs, using a limited set of markers [22,25,30,31,40,43,55,56,57,58,59,60,61,62]. However, most models are based on blood samples, which are the most validated sample type, as seen in independent studies. In forensic work, other tissues can also be important. Additionally, forensic models should work with the DNA methylation analysis technology found in routine laboratories. There are few useful tools that provide both mathematical models and validated lab protocols.

Concerning the genes and CpG regions being studied, numerous studies concentrate on only a small set of genes or investigate one specific gene along with particular CpG regions. In this research, we created a tailored panel of DNA methylation indicators, which included 610 CpG regions from 12 genes linked to aging spread across 135 amplicons, merging significant age-related loci into one test. Evaluating these important genes in a unified assay will broaden opportunities related to laboratory testing, such as creating independent mathematical models that predict outcomes for distinct somatic tissues.

Importantly, the exploration of additional CpG regions within the amplicons, as utilized in this investigation, offers a wider perspective on how a gene relates to aging mechanisms and allows for the recognition of the most significantly associated and consistent CpG regions, which aids in advancing resources for predicting epigenetic age.

AmpliSeq-Ion Torrent S5 represents a proven technology that allows for the effective and scalable examination of numerous target CpG sites, accommodating various DNA input amounts that could be relevant in forensic applications (10–100 ng). Additionally, the incorporated bioinformatics framework on the Ion Torrent server, featuring tailored plugins for methylation assessment like methylation-analysis, facilitates the handling of the data. Utilizing an automated platform (Ion Chef) for the preparation of libraries with kits formulated for human identification and medical diagnostics enhances workflow productivity and reduces contamination dangers.

Ion AmpliSeq technology utilized for focused DNA methylation evaluation has been incorporated in additional research efforts. For example, Fabrizio and colleagues (2022) introduced a new targeted next-generation sequencing framework for examining methylation, named OPERA_MET-A, which concurrently evaluates methylation levels at CpG sites in numerous genes associated with cancer [63]. Employing Ion AmpliSeq technology allowed the amplification of 155 regions, each averaging 125–175 bp in length, which encompassed a total of 1107 CpG sites spanning 18 genes linked to cancer.

Zunping Luo and associates (2020) established an automated system for designing primers for DNA methylation sequencing, simplifying the development of personalized methylation panels [64]. They crafted a comprehensive workflow taking three days, which included bisulfite treatment, library creation, template preparations, sequencing, and subsequent data analysis. The sequencing was conducted using the Ion GeneStudio S5 platform, while the bioinformatics evaluation was performed through a downloadable plugin that executes DNA alignment and methylation analysis, enabling the identification of amplicons on both the Watson and Crick strands.

The machine learning method presented here—which incorporates data-driven CpG selection, collinearity reduction and Elastic Net regression with natural splines—yielded a mean absolute error (MAE) of 4.42 years in repeated internal cross-validation (10-fold × 5 repeats) for the Watson-strand data matrix. This is a competitive value, particularly given the limited number of studies involving DNA methylation data generated using Ion AmpliSeq technology.

Watson-strand data consistently outperformed both Crick and combined Watson + Crick approaches across all evaluation criteria (CV MAE, nested-CV MAE, training correlation). This may reflect the higher signal-to-noise ratio of the Watson strand for certain CpG loci—notably KLF14 (Watson ρ = 0.67 vs. Crick ρ = 0.23) and EDARADD (Watson: limited reads; Crick: ρ = −0.72). The combined approach dilutes strong unidirectional signals with noisier opposite-strand measurements. This finding suggests that specific training using data obtained via Ion AmpliSeq sequencing enables the capture of technical and biological patterns specific to this platform, thereby avoiding the need for complex cross-platform corrections. This aspect is becoming increasingly important given the rise of MPS/NGS technologies in forensic genetics [23,65].

Among the six algorithms compared (Elastic Net, Ridge, LASSO, Random Forest, SVM and XGBoost), Elastic Net achieved the best performance for the Watson matrix, which was selected for the final model. In the Crick and Watson + Crick matrices, XGBoost and SVM showed marginally lower MAE values, respectively. However, the differences between the best-performing algorithms were small and generally overlapped within approximately one standard error. Elastic Net was therefore selected because it combined competitive performance with interpretability, stability under multicollinearity and parsimony. This choice is consistent with previous work reporting excellent performance of penalised linear models in epigenetic age prediction [1,15,40,43]. Vidaki et al. [1] and Aliferi et al. [15] had already shown that, although complex algorithms such as neural networks can capture non-linear relationships, their performance depends critically on sample size and data complexity. In moderate-sized cohorts, such as the one analysed here (n ≈ 110), regularised linear models offer significant advantages in terms of interpretability, stability and generalisation ability. The effectiveness of Elastic Net likely stems from its ability to handle multicollinearity among highly correlated CpGs, a phenomenon widely described in epigenetic clocks [66,67]. Furthermore, the combination of penalties allows biologically relevant variables to be retained whilst simultaneously avoiding overfitting, a common limitation in forensic studies with moderate sample sizes [28,42].

Watson’s final 5-CpG model encompasses five genes (C1orf132, ELOVL2, KLF14, PDE4C, TRIM59) with established roles in epigenetic ageing [22,25,31,56,57]. In particular, ELOVL2 has been repeatedly identified as one of the most robust predictors of age across multiple tissues and populations [22,31,57,59]. The application of natural splines to CpGs exhibiting non-linear age trajectories (e.g., C1orf132, KLF14) improved model flexibility without sacrificing parsimony, consistent with the known sigmoidal methylation kinetics at these loci. Elastic Net was selected as the definitive model because it combines competitive accuracy on the chosen Watson matrix with coefficient-level interpretability, stability under multicollinearity and parsimony—properties that are decisive for a forensic, reportable model.

Three lines of evidence support the robustness of the model: (i) nested cross-validation (MAE = 4.70) yielded estimates close to those of standard 10-fold cross-validation (MAE = 4.42), indicating minimal optimistic bias; (ii) the stability of CpG selection was high, with core representatives appearing in 80–100% of the out-of-fold samples; (iii) the negligible systematic bias across age groups suggests that the model does not suffer from age-dependent prediction drift. This aspect is particularly relevant given that several studies have highlighted the risk of overfitting in forensic epigenetic models [28,42,68]. The observed stability suggests that the model captures consistent biological signals that are independent of specific partitions of the training set.

In this study, the model based on the Watson strand data set consistently outperformed the Crick strand data and the combined model. Although most methylation studies assume functional equivalence between strands, our results suggest that there may be significant differences in signal quality, read depth or alignment efficiency depending on the sequencing orientation. This phenomenon could be related to technical biases inherent in technologies based on sodium bisulphite conversion and amplicon sequencing [63,64,69]. Therefore, this study allows us to examine the feasibility of incorporating DNA methylation data from the Watson and Crick strands into age prediction models, which highlights the need for further validation studies.

The prediction values obtained in this study were compared with those achieved in similar studies, taking into account criteria such as detection technique, number of genes and CpG sites analysed, number of samples used, and the error metric reported by each original publication. In this study, model performance is reported using the mean absolute error (MAE), defined as the mean of the absolute differences between predicted and chronological age. Some previous studies report MAD; because this term may refer to variability around a central value and is not necessarily equivalent to MAE, the original metric label used by each study has been retained in Table 2. Consequently, cross-study comparisons involving MAE and MAD should be interpreted as descriptive rather than strictly interchangeable.

From the table above, the following aspects can be highlighted as contributing to existing knowledge in the analysis of DNA methylation for forensic purposes, specifically age estimation: (i) The use of AmpliSeq-Ion Torrent technology is a valid alternative that should be considered in future studies; the more results that become available regarding its use, the greater our understanding of its strengths and areas for improvement will be. The present results suggest that specific training on Ion AmpliSeq allows for the capture of technical and biological patterns specific to this platform, avoiding the need for complex cross-platform corrections. This aspect is becoming increasingly relevant given the rise of MPS/NGS technologies in forensic genetics [23,65], particularly within the VISAGE ecosystem [13,54,74,75]. (ii) The use of customised DNA methylation marker panels via amplicons will allow for the exploration of a greater number of age-related genes and CpG sites. (iii) The use of DNA methylation levels obtained from the Watson and Crick strands, as well as their combination, is an alternative that can be utilised in age prediction models, for which further studies will be necessary.

Several limitations should be noted: (i) The sample size (n = 111 for Watson) is moderate and limits the power to detect subtle non-linearities or sex-specific effects. (ii) All samples were collected from a single population (Spanish adults aged 18–67), and external validation in independent cohorts is required; recent studies have shown that population factors, genetic ancestry and environmental exposures may partially influence age-related methylation patterns [76,77]. Fleckhaus et al. [77] observed moderate differences between European and Middle Eastern populations, whilst Piniewska-Róg et al. [76] reported effects of chronic alcohol abuse on epigenetic age prediction. These findings underscore the need for multicentre and multiethnic validations. (iii) The Qubit dsDNA HS assay quantifies total double-stranded DNA and is not human-specific; in buccal swabs a variable fraction is bacterial, so the true human input may be below the nominal 100 ng and the bisulfite conversion yield cannot be strictly referred to human DNA only. Importantly, only reads aligned to the human bisulfite genome (Bismark/GRCh38) contribute to the methylation calls, and the 50-read-per-CpG threshold plus the Lambda conversion control safeguard call quality; nonetheless, human-specific quantification (e.g., qPCR-based) is recommended for future work. (iv) The model was trained exclusively using DNA from buccal swabs, and its transferability to other tissues or degraded samples remains to be assessed due to the marked tissue specificity of many epigenetic clocks [2,17,55]. (v) The age range is restricted to adults (18–67 years), excluding paediatric and elderly populations where methylation dynamics may differ. (vi) Biological sex, comorbidities, and alcohol/tobacco consumption were not recorded and thus could not be modelled. These factors are known to influence DNA methylation and may affect age-prediction accuracy; their incorporation as covariates is proposed as future work.

The following areas for improvement can be identified: (i) external validation using an independent cohort; (ii) evaluation of the model’s performance with small amounts of DNA (1–10 ng) for forensic cases; (iii) the incorporation of sex as a covariate; (iv) the extension to degraded and mixed samples; and (v) the development of prediction intervals using quantile regression for the preparation of forensic reports.

## 4. Materials and Methods

### 4.1. Sampling

A total of 152 buccal swab samples were collected from volunteer participants (80 women and 72 men) aged between 18 and 67 years (mean 42.10 years ± SD 15.15). All participants were informed about the objectives of the research, the anonymous handling of samples, their storage, and the confidential processing of personal data and ancillary information before sample collection. Volunteers who agreed to participate provided written informed consent for collection of biological samples. This study was approved by the Human Research Ethics Committee of University of Granada, Spain, under certificate No. 3322/CEIH/2023, dated 2 March 2023.

Buccal swab samples were collected following a protocol that was established by the Genetic Identification Laboratory of University of Granada (LABIGEN), ensuring sterility and biosafety conditions at all times. Dry rayon swabs were used in APTACA 2160/SG tubes (APTACA S.p.A., Canelli, Italy).

Age groups were defined in 10-year intervals, with the year 2023 serving as the reference point to determine the chronological age of participants at the time of sampling, as shown in Table 3.

### 4.2. DNA Extraction and Quantification

Extraction and purification of DNA from the buccal swab samples were performed using an automated procedure on the QIAcube system (QIAGEN, Hilden, Germany), in conjunction with the QIAamp DNA Investigator Kit (QIAGEN, Hilden, Germany). A total of 100 μL of purified DNA extract was obtained. Total DNA extracts were quantified using the Qubit™ dsDNA HS Assay Kit (Invitrogen, Thermo Fisher Scientific, Waltham, MA, USA) on a Qubit 4 fluorometer (Thermo Fisher Scientific, Waltham, MA, USA), following the manufacturer’s approved procedures.

### 4.3. Sodium Bisulfite Conversion

The MethylCode™ Bisulfite Conversion Kit (Thermo Fisher Scientific, Waltham, MA, USA) was used. All samples were normalized to 100 ng as the starting amount of DNA for conversion, per the manufacturer’s recommendations.

The methylation level was calculated as the ratio of methylated reads to the total number of methylated and unmethylated reads and was expressed as a percentage. To determine bisulfite conversion rates, 0.005016 ng of unmethylated Lambda DNA (Promega, Madison, WI, USA) was added to each DNA extract before conversion.

Thermo Fisher Scientific Methylated Human Genomic DNA (Thermo Fisher Scientific, Waltham, MA, USA; CpG Methylated Jurkat Genomic DNA, 100 ng/μL) was included as a quality control sample for the validation process, with 0.005016 ng of unmethylated Lambda DNA (Promega, Madison, WI, USA) also added to this control.

Conversion with sodium bisulfite was performed using 100 ng of initial DNA. From this amount of DNA, dilutions were prepared at a concentration of 4 ng/μL for a total volume of 25 μL. In samples where DNA concentrations were lower than 4 ng/μL, 25 μL of the original undiluted DNA extract was processed. Thus, all samples (diluted and undiluted) for conversion had an initial DNA content of at least 10 ng, meeting the input range specified in the kit (10–100 ng).

The conversion rate per sample was assessed following the Thermo Fisher Scientific MAN0017662 manual guidelines. According to the manufacturer’s recommendations, conversion rates >99% are expected, although conversion rates between 95% and 100% were also considered to be acceptable, as indicated in [69].

Following sodium bisulfite conversion, 10 μL of converted and purified DNA extract was obtained and stored in LoBind tubes at −20 °C.

### 4.4. Selection of Age-Related Genes and CpG Sites

A literature review was conducted to identify candidate genes with a methylation status that was associated with age. Genes were selected based on the following criteria: (i) association of gene methylation with aging in multiple independent studies, (ii) relevance across different tissue types, and (iii) integration into existing models, allowing for their validation. Twelve genes were chosen per these criteria: ASPA, C1orf132 (or MIR29B2CHG), CCDC102B, EDARADD, ELOVL2, FHL2, ITGA2B, KLF14, PDE4C, SCGN, TRIM59, and ZNF423 [14,23,24,28,29,32,42,54,55,56,78].

For each of these 12 genes, CpG sites were selected according to the following criteria: (i) they have been investigated in the available literature; (ii) they are located in exonic and promoter regions to evaluate age-associated hypomethylation and hypermethylation processes; and (iii) they are clustered with additional CpG sites to maximize the available methylation data, facilitating future analyses of degraded or low-quality DNA samples.

Using the Ensembl genomic browser (https://www.ensembl.org/index.html, accessed on 31 July 2026), genomic regions between 180 and 900 bp were selected near the promoter regions of each gene, based on the GRCh38 human genome assembly. According to these criteria, a total of 649 detectable CpG sites were identified. Thermo Fisher Scientific (Waltham, MA, USA) was consulted to design a custom DNA methylation panel targeting the selected regions in the 12 genes of interest. The final panel included amplicons 125 to 175 bp in length, targeting the Watson and Crick strands where the design allowed. The resulting 610 CpG targets within the 12 selected genes were organized into two primer groups (Supplementary Material S1).

### 4.5. Custom Panel Amplification

Following the Thermo Fisher Scientific (Waltham, MA, USA) Bisulfite Methylation Library Production and Analysis recommendations using the Ion AmpliSeq™ Library Kit Plus (MAN0017662), 2 amplification reactions were run for each sample—1 per primer pool. Briefly, in a reaction tube, 4.5 μL 5× Ion AmpliSeq™ HiFi Mix and 12.5 μL nuclease-free water were combined. Subsequently, 1 μL of DNA (obtained from sodium bisulfite conversion, ≥10 ng) was added, resulting in a final volume of 18 μL (reaction mixture + DNA). From this mixture, 8 μL was split into 2 PCR tubes (1 for each primer pool), according to the manufacturer’s recommendations. Finally, 2 μL of the corresponding 5× primer pool was added to each PCR tube, yielding a final reaction volume of 10 μL per tube.

The PCR reaction was run on a Veriti thermal cycler (Applied Biosystems, Thermo Fisher Scientific, Waltham, MA, USA) under the following conditions: 99 °C for 2 min, followed by 28 cycles of 99 °C for 15 s and 60 °C for 4 min. On completion, the 10-μL amplification reactions were combined, resulting in a volume of approximately 20 μL per sample.

### 4.6. Partial Digestion of Amplicons

For each amplified sample, 2 μL of FuPa Reagent was added, yielding a final volume of approximately 22 μL. The digestion reaction was run on a Veriti thermal cycler (Applied Biosystems, Thermo Fisher Scientific, Waltham, MA, USA) under the following conditions: 50 °C for 10 min, 55 °C for 10 min, and 60 °C for 20 min.

### 4.7. Adapter Binding and Library Purification

Barcode adapters were ligated to amplicons per the Thermo Fisher Scientific manual MAN0017662 (Bisulfite Methylation Library Production and Analysis using the Ion AmpliSeq™ Library Kit Plus). Briefly, the following reagents were added sequentially to the tube that contained the digested amplicon reaction from the previous step: 4 µL of Switch Solution, 2 µL of diluted barcode adapter, and 2 µL of DNA ligase—resulting in a final volume of approximately 30 µL.

The adapter ligation reaction was run on a Veriti thermal cycler (Applied Biosystems, Thermo Fisher Scientific, Waltham, MA, USA) under the following conditions: 22 °C for 30 min, 68 °C for 5 min, and 72 °C for 5 min. Library purification was performed in 2 rounds using Agencourt™ AMPure XP (Beckman Coulter, Brea, CA, USA), following the instructions in MAN0017662, with some modifications: (i) 5 min of incubation at room temperature with Agencourt™ AMPure XP reagent, (ii) 5 min of incubation on a magnetic plate to separate beads, (iii) 2 min of drying at room temperature after discarding the 70% ethanol, and (iv) 5 min of incubation with low TE at room temperature.

### 4.8. Quantification of Libraries by Real-Time PCR

The purified libraries were quantified using the Ion Library TaqMan Quantitation Kit (Thermo Fisher Scientific, Waltham, MA, USA) on the QuantStudio S5 system (Applied Biosystems) under the following conditions: 50 °C for 2 min, 95 °C for 2 min, and 40 cycles of 95 °C for 15 s and 60 °C for 1 min.

Some libraries were quantified using the High-Sensitivity DNA Kit (Agilent Technologies, Santa Clara, CA, USA) on a Bioanalyzer 2100 instrument (Agilent Technologies, Santa Clara, CA, USA) to verify the size of the amplicons. The manufacturer’s recommendations were followed for preparing the reactions.

### 4.9. Reamplification of Libraries

A minimum threshold of 50 reads (sum of methylated and unmethylated reads per amplicon) was established as a criterion for accepting the quality of the quantification of DNA methylation [14]. Libraries that did not meet this threshold underwent a reamplification and were resequenced to recover as much information as possible, obviating the need to repeat amplification of the original methylation panel.

This procedure followed the recommendations in MAN0017003 D.0 Ion AmpliSeq™ Library Kit Plus (January 2024, Thermo Fisher Scientific, Waltham, MA, USA). Briefly, Agencourt™ AMPure™ XP was added at a 1:1 volume relative to the available library extract; after 5 min of incubation, the supernatant was discarded using a magnet. Next, 50 μL of 1× Library Amp Mix and 2 μL of 25× Library Amp Primers were added to the bead pellet. The reamplification reaction was performed on a Veriti thermal cycler (Applied Biosystems, Thermo Fisher Scientific, Waltham, MA, USA) under the following conditions: 98 °C for 2 min and 9 cycles of 98 °C for 15 s and 64 °C for 1 min. The reamplification extract was transferred to a new tube, purified using 1× Agencourt™ AMPure™ XP (~50 μL), and subjected to a final round of purification following the MAN0017662 instructions.

### 4.10. Template Preparation and Sequencing

Libraries were normalized to 50 pM, and 16 libraries per sequencing were pooled in equal volumes before preparation of the template. For libraries with results below 50 pM, 5 μL of undiluted library extract was used to prepare the library pool. Template creation and subsequent sequencing on the Ion GeneStudio S5 system (Thermo Fisher Scientific, Waltham, MA, USA) were performed using the ExT Ion 520™ and Ion 530™ Kit-Chef (A30670; Thermo Fisher Scientific, Waltham, MA, USA) following the manufacturer’s instructions (MAN0015805).

Among the quality parameters, the total number of reads (7–9 million), usable reads (>30%), percentage of chip loading (43–57%), test fragments (1–2%), and polyclonality (20–25%) were verified.

The sequencing results were analyzed using Ion Torrent Suite Server 5.10.1, with the methylation_analysis plugin. This plugin used a modified version of Bismark to align reads to an in-silico bisulfite genome (GRCh38_Lambda). In this study, the terms Watson and Crick are used as operational strand annotations derived from the reference genome and the strand-specific bisulfite-alignment output. The Watson strand corresponds to the original top/reference forward (+) strand of each target region, whereas the Crick strand corresponds to the original bottom/reference reverse-complement (−) strand. Thus, these labels refer to the genomic strand on which the CpG methylation call was assigned by the analysis workflow, not to coding versus template status of a gene. Methylated (ME) and unmethylated (UM) reads were counted separately for target cytosines assigned to each strand, and beta (β) methylation values were calculated as ME/(ME + UM). The final methylation call was made using a minimum threshold of 50 reads, calculated as the sum of methylated and unmethylated reads per amplicon for each CpG site. CpG sites that failed to meet this criterion were excluded from the corresponding analysis matrix.

### 4.11. Development of the Age Prediction Model

#### 4.11.1. Preprocessing and Quality Control of Raw Data

Raw per-CpG methylation data were extracted from Ion Torrent output files and organized into a unified data frame (one row per CpG × sample × strand observation). The following preprocessing and quality-control parameters were taken into account: COV_THRESHOLD: 50 minimum reads per record (sum of methylated and non-methylated reads); DEDUPLICATION: exact duplicate records per sample, CpG, and strand were removed; STRAND RESOLUTION: three parallel methylation matrices were constructed according to the operational strand definition described above: Watson strand only (dat_W; CpGs assigned to the original top/reference forward strand), Crick strand only (dat_C; CpGs assigned to the original bottom/reference reverse-complement strand), and Watson + Crick (dat_WC; CpG-level average of Watson and Crick values when both strand-specific measurements were available). For the combined WC matrix, values were set to missing when the absolute Watson–Crick difference exceeded 10 percentage points, as this was considered a marker of possible strand-dependent noise or asymmetric conversion. CPG-LEVEL FILTERING: CpG sites with >20% missing values across samples were excluded; SAMPLE-LEVEL FILTERING: samples with >20% missing CpGs were removed; LOW-VARIANCE FILTERING: CpG sites with variance <1 were discarded as uninformative; OUTLIER EXCLUSION: three samples (048-C, 088-E and 145-A) were excluded based on global QC diagnostics. After QC, the final analytical datasets comprised 111 samples for Watson, 109 for Crick and 110 for Watson + Crick, with complete methylation profiles for the retained CpG sets. This approach allowed us to test whether strand-specific methylation estimates carried different age-predictive information.

An exploratory data analysis was performed to detect outliers and evaluate which CpGs show the greatest association with the age group before moving on to predictive modeling. The software used was RStudio version 2026.01.2 Build 418 (Posit Software, PBC, Boston, MA, USA).

#### 4.11.2. Univariate Metric Calculation

For each CpG site, three univariate metrics were computed against chronological age: Spearman correlation coefficient (|ρ| ≥ 0.50), interquartile range of methylation values (IQR ≥ 5 percentage points), and coefficient of determination from simple linear regression (R^2^ ≥ 0.25). CpG sites meeting all three thresholds and with ≥80% non-missing observations were retained as candidates.

#### 4.11.3. Collinearity Reduction

Among candidate CpGs, pairwise Pearson correlation coefficients were computed. When two CpGs showed |r| > 0.85, the one with lower |ρ| with age was removed using a greedy iterative algorithm.

#### 4.11.4. Multivariate Feature Selection

Elastic Net regression (glmnet R package v4.1) was applied to the filtered CpG set with chronological age as the response variable. A grid of mixing parameters α ∈ {0.25, 0.50, 0.75, 1.00} was evaluated via 10-fold internal cross-validation, selecting the regularization parameter λ at the one-standard-error rule (lambda.1se) for parsimony. As a complementary approach, stepwise AIC selection (MASS package) was performed. CpGs with non-zero Elastic Net coefficients formed the multivariate candidate set.

#### 4.11.5. Cross-Validation Strategy

To prevent information leakage, a full-pipeline cross-validation scheme was implemented: within each fold, the entire feature-selection process (univariate screening → collinearity purge → Elastic Net fitting) was re-executed on training data only. Two schemes were evaluated: (i) leave-one-out cross-validation (LOO-CV) and (ii) 10-fold CV repeated 5 times. Performance was assessed by mean absolute error (MAE), root mean square error (RMSE), and coefficient of determination (R^2^).

#### 4.11.6. Algorithm Comparison

Six regression algorithms were compared under identical 10-fold × 5-repeat CV conditions using the tidymodels framework (R v4.3): Elastic Net, Ridge, LASSO, Random Forest (ranger), Support Vector Machine with radial kernel (kernlab), and Gradient Boosted Trees (XGBoost). For each algorithm, 20 random hyperparameter configurations were evaluated, optimizing MAE as the primary metric. Variable importance was extracted using the vip package.

#### 4.11.7. Sensitivity Analysis of Feature-Selection Thresholds

Nine scenarios were evaluated, varying ρ thresholds (0.50–0.65), α mode (free grid vs. LASSO only), and λ criterion (lambda.1se vs. lambda.min). LOO-CV MAE was computed for each scenario to identify the parsimony–accuracy trade-off.

#### 4.11.8. Non-Linearity Assessment and Natural Splines

For each candidate CpG, a linear model (CpG ~ age) was compared to a natural cubic spline model (ns(CpG, df = 3) ~ age) using ΔAIC and ANOVA F-test with Benjamini–Hochberg correction. CpGs with ΔAIC > 10 and adjusted *p* < 0.10 were classified as non-linear and modeled with natural spline basis expansions (df = 3) in subsequent steps.

#### 4.11.9. Representative CpG Selection (One per Gene)

To maximize parsimony, a single representative CpG per gene was selected using a composite score: 0.70 × normalized |Elastic Net coefficient| + 0.30 × normalized ΔAIC (non-linearity signal). Nested cross-validation (5 outer × 5 inner folds) was used to obtain an unbiased estimate of generalization error for the reduced panel. Dispensability analysis (leave-one-CpG-out) quantified the contribution of each representative.

#### 4.11.10. Final Age Prediction Model

The definitive model was trained using the representative CpGs (5 for Watson, 6 for Crick, 7 for Watson + Crick), applying natural splines (df = 3) only to CpGs with confirmed non-linearity. The modeling workflow (tidymodels) included median imputation, near-zero-variance filtering, predictor normalization, and Elastic Net regression. Hyperparameters (penalty and mixture) were tuned via 10-fold CV × 5 repeats, optimizing MAE. The final model was fitted on the complete training set.

#### 4.11.11. Software and Reproducibility

All statistical analyses were conducted in R v4.3.0 using the following R packages: tidyverse, tidymodels, glmnet, splines, MASS, ranger, kernlab, xgboost, vip, quantreg, flextable, and patchwork. The random seed was fixed at 2024. Analysis scripts are available as reproducible R Markdown documents.

## 5. Conclusions

This study demonstrates that age estimation based on DNA methylation can be significantly improved using models trained specifically on data obtained via Ion AmpliSeq and machine learning strategies based on Elastic Net. The model developed achieved high accuracy (MAE = 4.42 years) for the final Watson model comprising 5 CpGs, covering five genes (C1orf132, ELOVL2, KLF14, PDE4C, TRIM59). The research emphasizes the significance of strand specificity in targeted methylation studies, revealing that analyses based on the Watson strand consistently yield better results than those based on the Crick strand or combined data. This observation indicates that upcoming methodological advancements should explicitly take into account how sequencing orientation affects predictive accuracy.

The results show that combining data-driven CpG selection, collinearity reduction and penalised regularisation enables the construction of parsimonious and robust models, whilst maintaining high predictive power using just five epigenetic markers. The incorporation of natural splines for CpGs with non-linear methylation trajectories further improved the model’s flexibility without unduly increasing its complexity.

The proposed method marks a crucial advancement toward the regular use of age-estimation tools that utilize methylation analysis within forensic genetics. Nonetheless, it will be important to conduct external validations in separate populations, enhance the sample size for each year of age, expand the analysed age range, and assess the performance of the model in real-world forensic cases, including scenarios with degraded DNA, limited samples, and mixed samples. These forthcoming validations are vital for establishing the model’s practical effectiveness and reliability.

## Figures and Tables

**Table 1:** Final dataset dimensions after quality control (QC).

Matrix	Samples After QC	CpGs After Filtering	Coverage (Mean Reads/CpG)
Watson (W)	111	~280	181.6 ± 60.5 k
Crick (C)	109	~260	181.6 ± 60.5 k
Watson + Crick (WC)	110	~240	181.6 ± 60.5 k

**Table 2:** Age prediction accuracy.

Ref	Detection Technique	No. Genes	No. CpG	Sample Size	Prediction Accuracy Years
[30]	Pyrosequencing	4	8	50	3.32 (MAD)
[70]	Pyrosequencing	5	5	55	4.3 (MAD) 5.2 (MAD) 4.66 (MAD)
[31]	SNaPshot	5	5	100	3.82 (MAD)
[71]	Pyrosequencing SNaPshot	3	3	95	5.11 (MAD) 5.16 (MAD)
[54]	MiSeq	5	9	111	2.5 (MAE)
[72]	SNaPshot	7	7	184	3.54 (MAE)
[29]	SNaPshot	8	8	230	4.69 (MAD)
[73]	Pyrosequencing	3	8	461	4.39 (MAE)
[60]	SNaPshot	5	5	60	3.49 (MAD)
[71]	Pyrosequencing	1	9	83	5.04 (MAE)
[51]	SNaPshot MiSeq FGx	5	5	37	5.75 (MAE)
[51]	SNaPshot MiSeq FGx	5	9	37	3.55 (MAE)
[33]	Pyrosequencing	1	9	83	5.04 (MAE)
[34]	SNaPshot	9	10	216	3.19 (MAE)
[35]	SNaPshot	5	9	36	5.75 (MAE)
Present study	Ion AmpliSeq/ Ion Torrent S5	12	7	110	5.08 (W + C)
Present study	Ion AmpliSeq/ Ion Torrent S5	12	6	109	4.75 (Crick)
Present study	Ion AmpliSeq/ Ion Torrent S5	12	5	111	4.42 (Watson)

Note: The prediction-accuracy metric is reported as stated in each original publication. MAE refers to the mean absolute error between predicted and chronological age. MAD values reported in the literature are retained with their original label; because MAE and MAD are not necessarily interchangeable, comparisons across different metrics should be interpreted descriptively.

**Table 3:** Establishment of age groups for this research.

Group	Age Range in Years	Year of Birth
A	18–27	1996–2005
B	28–37	1986–1995
C	38–47	1976–1985
D	48–57	1966–1975
E	58–67	1956–1965

## Data Availability

The datasets that were used and/or analysed during this study are available upon reasonable request from the corresponding author.

## Supplementary Materials

The following supporting information can be downloaded at: https://www.mdpi.com/article/10.3390/ijms27157062/s1. Refs [79,80,81,82,83] are cited in Supplementary Material S2.

## Abbreviations

The following abbreviations are used in this manuscript:

CpG	Cytosine followed by Guanine nucleotide
DNA	Deoxyribonucleic acid
MAE	Mean absolute error
PCR	Polymerase chain reaction
RMSE	Root mean square error
