# Supplementary material for: Development of a Model for Predicting Chronological Age Based on DNA Methylation Data from Buccal Swabs Using Ion Torrent S5 Technology
Source: Int J Mol Sci. 2026 Aug 6;27(15):7062. doi: 10.3390/ijms27157062 (PMC13466995; doi:10.3390/ijms27157062)
Supplement: Supplementary file 1 [file ijms-27-07062-s001.zip › SM1_Features_Custom_DNA_methylation_panel_WG00651_MET_V2.pdf]

Supplementary Material S1

Features Custom DNA methylation panel WG00651_MET_V2

1. CpG sites covered and not covered in the panel design

Item	Gene_Symbol	Chromosome	Posicion CpG	Gen-CpG	Status
1	r72	chr1	207823591	C1orf132_CpG-1	Proposal
2	r73	chr1	207823616	C1orf132_CpG-2	Proposal
3	r74	chr1	207823637	C1orf132_CpG-3	Proposal
4	r75	chr1	207823657	C1orf132_CpG-4	Proposal
5	r76	chr1	207823660	C1orf132_CpG-5	Proposal
**6**	**r1**	**chr1**	**207823672**	**C1orf132_CpG-6**	**Mandatory**
**7**	**r2**	**chr1**	**207823675**	**C1orf132_CpG-7**	**Mandatory**
**8**	**r3**	**chr1**	**207823681**	**C1orf132_CpG-8**	**Mandatory**
**9**	**r4**	**chr1**	**207823702**	**C1orf132_CpG-9**	**Desirable**
**10**	**r5**	**chr1**	**207823705**	**C1orf132_CpG-10**	**Desirable**
**11**	**r6**	**chr1**	**207823715**	**C1orf132_CpG-11**	**Desirable**
12	r77	chr1	207823723	C1orf132_CpG-12	Proposal
13	r78	chr1	207823760	C1orf132_CpG-13	Proposal
14	r79	chr1	207823763	C1orf132_CpG-14	Proposal
15	r80	chr1	207823821	C1orf132_CpG-15	Proposal
16	r81	chr1	207823828	C1orf132_CpG-16	Proposal
17	r82	chr1	207823832	C1orf132_CpG-17	Proposal
18	r83	chr1	207823844	C1orf132_CpG-18	Proposal
19	r84	chr1	207823901	C1orf132_CpG-19	Proposal
20	r85	chr1	207823934	C1orf132_CpG-20	Proposal
21	r86	chr1	207823986	C1orf132_CpG-21	Proposal
22	r87	chr1	207824008	C1orf132_CpG-22	Proposal
23	r88	chr1	207824011	C1orf132_CpG-23	Proposal
24	r89	chr1	207824026	C1orf132_CpG-24	Proposal
25	r90	chr1	207824030	C1orf132_CpG-25	Proposal
26	r91	chr1	207824085	C1orf132_CpG-26	Proposal
**27**	**r7**	**chr1**	**236394370**	**EDARADD_CpG-1**	**Mandatory**
**28**	**r8**	**chr1**	**236394382**	**EDARADD_CpG-2**	**Mandatory**
**29**	**r9**	**chr1**	**236394441**	**EDARADD_CpG-3**	**Desirable**
30	r92	chr1	236394458	EDARADD_CpG-4	Proposal
31	r93	chr1	236395177	EDARADD_CpG-5	Proposal
32	r94	chr1	236395198	EDARADD_CpG-6	Proposal
33	r95	chr1	236395202	EDARADD_CpG-7	Proposal
34	r96	chr1	236395214	EDARADD_CpG-8	Proposal
35	r97	chr1	236395218	EDARADD_CpG-9	Proposal
36	r98	chr1	236395225	EDARADD_CpG-10	Proposal
37	r99	chr1	236395238	EDARADD_CpG-11	Proposal
38	r100	chr1	236395248	EDARADD_CpG-12	Proposal
39	r101	chr1	236395260	EDARADD_CpG-13	Proposal
40	r102	chr1	236395269	EDARADD_CpG-14	Proposal
41	r103	chr1	236395278	EDARADD_CpG-15	Proposal

42	r104	chr1	236395280	EDARADD_CpG-16	Proposal
43	r105	chr1	236395282	EDARADD_CpG-17	Proposal
44	r106	chr1	236395291	EDARADD_CpG-18	Proposal
45	r107	chr1	236395300	EDARADD_CpG-19	Proposal
46	r108	chr1	236395304	EDARADD_CpG-20	Proposal
47	r109	chr1	236395313	EDARADD_CpG-21	Proposal
48	r110	chr1	236395321	EDARADD_CpG-22	Proposal
49	r111	chr1	236395328	EDARADD_CpG-23	Proposal
50	r112	chr1	236395339	EDARADD_CpG-24	Proposal
51	r113	chr1	236395344	EDARADD_CpG-25	Proposal
52	r114	chr1	236395346	EDARADD_CpG-26	Proposal
53	r115	chr1	236395354	EDARADD_CpG-27	Proposal
54	r116	chr1	236395361	EDARADD_CpG-28	Proposal
55	r117	chr1	236395366	EDARADD_CpG-29	Proposal
56	r118	chr1	236395368	EDARADD_CpG-30	Proposal
57	r119	chr1	236395387	EDARADD_CpG-31	Proposal
58	r120	chr1	236395394	EDARADD_CpG-32	Proposal
59	r121	chr1	236395396	EDARADD_CpG-33	Proposal
60	r122	chr1	236395401	EDARADD_CpG-34	Proposal
61	r123	chr1	236395440	EDARADD_CpG-35	Proposal
62	r124	chr1	236395451	EDARADD_CpG-36	Proposal
63	r125	chr1	236395482	EDARADD_CpG-37	Proposal
64	r126	chr1	236395498	EDARADD_CpG-38	Proposal
65	r127	chr1	236395500	EDARADD_CpG-39	Proposal
66	r128	chr1	236395510	EDARADD_CpG-40	Proposal
67	r129	chr1	236395524	EDARADD_CpG-41	Proposal
68	r130	chr1	236395526	EDARADD_CpG-42	Proposal
69	r131	chr1	236395529	EDARADD_CpG-43	Proposal
70	r132	chr1	236395535	EDARADD_CpG-44	Proposal
71	r133	chr1	236395553	EDARADD_CpG-45	Proposal
72	r134	chr1	236395563	EDARADD_CpG-46	Proposal
73	r135	chr1	236395572	EDARADD_CpG-47	Proposal
74	r136	chr1	236395574	EDARADD_CpG-48	Proposal
75	r137	chr1	236395579	EDARADD_CpG-49	Proposal
76	r138	chr1	236395596	EDARADD_CpG-50	Proposal
77	r139	chr1	236395600	EDARADD_CpG-51	Proposal
78	r140	chr1	236395604	EDARADD_CpG-52	Proposal
79	r141	chr1	236395608	EDARADD_CpG-53	Proposal
80	r142	chr1	236395623	EDARADD_CpG-54	Proposal
81	r143	chr1	236395626	EDARADD_CpG-55	Proposal
82	r144	chr1	236395640	EDARADD_CpG-56	Proposal
83	r145	chr1	236395644	EDARADD_CpG-57	Proposal
84	r146	chr1	236395654	EDARADD_CpG-58	Proposal
85	r147	chr1	236395656	EDARADD_CpG-59	Proposal
86	r148	chr1	236395675	EDARADD_CpG-60	Proposal
87	r149	chr1	236395678	EDARADD_CpG-61	Proposal
88	r150	chr1	236395680	EDARADD_CpG-62	Proposal
89	r151	chr1	236395684	EDARADD_CpG-63	Proposal
90	r152	chr1	236395695	EDARADD_CpG-64	Proposal

91	r153	chr1	236395702	EDARADD_CpG-65	Proposal
92	r154	chr1	236395704	EDARADD_CpG-66	Proposal
93	r155	chr1	236395709	EDARADD_CpG-67	Proposal
94	r156	chr1	236395717	EDARADD_CpG-68	Proposal
95	r157	chr1	236395724	EDARADD_CpG-69	Proposal
96	r158	chr1	236395728	EDARADD_CpG-70	Proposal
97	r159	chr1	236395737	EDARADD_CpG-71	Proposal
98	r160	chr1	236395748	EDARADD_CpG-72	Proposal
99	r161	chr1	236395754	EDARADD_CpG-73	Proposal
100	r162	chr1	236395761	EDARADD_CpG-74	Proposal
101	r163	chr1	236395764	EDARADD_CpG-75	Proposal
102	r164	chr1	236395771	EDARADD_CpG-76	Proposal
103	r650	chr1	236395138	EDARADD_CpG-77	Detected
104	r651	chr1	236395160	EDARADD_CpG-78	Detected
105	r652	chr1	236395165	EDARADD_CpG-79	Detected
106	r653	chr1	236395167	EDARADD_CpG-80	Detected
107	r654	chr1	236395782	EDARADD_CpG-81	Detected
108	r655	chr1	236395795	EDARADD_CpG-82	Detected
109	r656	chr1	236395804	EDARADD_CpG-83	Detected
110	r657	chr1	236395813	EDARADD_CpG-84	Detected
111	r658	chr1	236395819	EDARADD_CpG-85	Detected
112	r659	chr1	236395823	EDARADD_CpG-86	Detected
113	r165	chr2	105398814	FHL2_CpG-1	Proposal
114	r166	chr2	105398857	FHL2_CpG-2	Proposal
115	r167	chr2	105398884	FHL2_CpG-3	Proposal
116	r168	chr2	105398889	FHL2_CpG-4	Proposal
117	r169	chr2	105398895	FHL2_CpG-5	Proposal
118	r170	chr2	105398908	FHL2_CpG-6	Proposal
119	r171	chr2	105398931	FHL2_CpG-7	Proposal
120	r172	chr2	105398942	FHL2_CpG-8	Proposal
121	r173	chr2	105398947	FHL2_CpG-9	Proposal
122	r174	chr2	105398969	FHL2_CpG-10	Proposal
123	r175	chr2	105398975	FHL2_CpG-11	Proposal
124	r176	chr2	105398980	FHL2_CpG-12	Proposal
125	r177	chr2	105398985	FHL2_CpG-13	Proposal
126	r178	chr2	105398995	FHL2_CpG-14	Proposal
127	r179	chr2	105398999	FHL2_CpG-15	Proposal
128	r180	chr2	105399012	FHL2_CpG-16	Proposal
129	r181	chr2	105399014	FHL2_CpG-17	Proposal
130	r182	chr2	105399018	FHL2_CpG-18	Proposal
131	r183	chr2	105399031	FHL2_CpG-19	Proposal
132	r184	chr2	105399037	FHL2_CpG-20	Proposal
133	r185	chr2	105399043	FHL2_CpG-21	Proposal
134	r186	chr2	105399047	FHL2_CpG-22	Proposal
135	r187	chr2	105399056	FHL2_CpG-23	Proposal
136	r188	chr2	105399061	FHL2_CpG-24	Proposal
137	r189	chr2	105399070	FHL2_CpG-25	Proposal
138	r190	chr2	105399076	FHL2_CpG-26	Proposal

139	r191	chr2	105399086	FHL2_CpG-27	Proposal
140	r192	chr2	105399089	FHL2_CpG-28	Proposal
141	r193	chr2	105399118	FHL2_CpG-29	Proposal
142	r194	chr2	105399123	FHL2_CpG-30	Proposal
143	r195	chr2	105399128	FHL2_CpG-31	Proposal
144	r196	chr2	105399137	FHL2_CpG-32	Proposal
145	r197	chr2	105399141	FHL2_CpG-33	Proposal
146	r198	chr2	105399148	FHL2_CpG-34	Proposal
147	r199	chr2	105399150	FHL2_CpG-35	Proposal
148	r200	chr2	105399157	FHL2_CpG-36	Proposal
149	r201	chr2	105399161	FHL2_CpG-37	Proposal
150	r202	chr2	105399174	FHL2_CpG-38	Proposal
151	r203	chr2	105399176	FHL2_CpG-39	Proposal
152	r204	chr2	105399183	FHL2_CpG-40	Proposal
153	r205	chr2	105399191	FHL2_CpG-41	Proposal
154	r206	chr2	105399196	FHL2_CpG-42	Proposal
155	r207	chr2	105399203	FHL2_CpG-43	Proposal
156	r208	chr2	105399206	FHL2_CpG-44	Proposal
157	r209	chr2	105399212	FHL2_CpG-45	Proposal
158	r210	chr2	105399217	FHL2_CpG-46	Proposal
159	r211	chr2	105399248	FHL2_CpG-47	Proposal
160	r212	chr2	105399257	FHL2_CpG-48	Proposal
**161**	**r10**	**chr2**	**105399282**	**FHL2_CpG-49**	**Mandatory**
**162**	**r11**	**chr2**	**105399288**	**FHL2_CpG-50**	**Mandatory**
**163**	**r12**	**chr2**	**105399291**	**FHL2_CpG-51**	**Mandatory**
**164**	**r13**	**chr2**	**105399297**	**FHL2_CpG-52**	**Mandatory**
**165**	**r14**	**chr2**	**105399300**	**FHL2_CpG-53**	**Mandatory**
**166**	**r15**	**chr2**	**105399310**	**FHL2_CpG-54**	**Mandatory**
**167**	**r16**	**chr2**	**105399314**	**FHL2_CpG-55**	**Mandatory**
**168**	**r17**	**chr2**	**105399316**	**FHL2_CpG-56**	**Mandatory**
**169**	**r18**	**chr2**	**105399323**	**FHL2_CpG-57**	**Mandatory**
**170**	**r19**	**chr2**	**105399327**	**FHL2_CpG-58**	**Mandatory**
171	r213	chr2	105399338	FHL2_CpG-59	Proposal
172	r214	chr2	105399340	FHL2_CpG-60	Proposal
173	r215	chr2	105399360	FHL2_CpG-61	Proposal
174	r216	chr2	105399363	FHL2_CpG-62	Proposal
175	r217	chr2	105399388	FHL2_CpG-63	Proposal
176	r218	chr2	105399412	FHL2_CpG-64	Proposal
177	r219	chr2	105399417	FHL2_CpG-65	Proposal
178	r220	chr2	105399426	FHL2_CpG-66	Proposal
179	r221	chr2	105399487	FHL2_CpG-67	Proposal
180	r222	chr2	105399521	FHL2_CpG-68	Proposal
181	r223	chr2	105399557	FHL2_CpG-69	Proposal
182	r224	chr2	105399567	FHL2_CpG-70	Proposal
183	r225	chr2	105399609	FHL2_CpG-71	Proposal
184	r226	chr2	105399653	FHL2_CpG-72	Proposal
185	r227	chr2	105399678	FHL2_CpG-73	Proposal
186	r660	chr2	105399700	FHL2_CpG-74	Detected

187	r661	chr2	105399711	FHL2_CpG-75	Detected
188	r662	chr2	105399716	FHL2_CpG-76	Detected
189	r663	chr2	105399722	FHL2_CpG-77	Detected
190	r228	chr3	160449690	TRIM59_CpG-1	Proposal
191	r229	chr3	160449696	TRIM59_CpG-2	Proposal
192	r230	chr3	160449717	TRIM59_CpG-3	Proposal
193	r231	chr3	160449719	TRIM59_CpG-4	Proposal
194	r232	chr3	160449730	TRIM59_CpG-5	Proposal
195	r233	chr3	160449752	TRIM59_CpG-6	Proposal
196	r234	chr3	160449763	TRIM59_CpG-7	Proposal
197	r235	chr3	160449768	TRIM59_CpG-8	Proposal
198	r236	chr3	160449779	TRIM59_CpG-9	Proposal
199	r237	chr3	160449793	TRIM59_CpG-10	Proposal
200	r238	chr3	160449798	TRIM59_CpG-11	Proposal
201	r239	chr3	160449806	TRIM59_CpG-12	Proposal
202	r240	chr3	160449821	TRIM59_CpG-13	Proposal
203	r241	chr3	160449844	TRIM59_CpG-14	Proposal
204	r242	chr3	160449848	TRIM59_CpG-15	Proposal
205	r243	chr3	160449856	TRIM59_CpG-16	Proposal
206	r244	chr3	160449858	TRIM59_CpG-17	Proposal
207	r245	chr3	160449866	TRIM59_CpG-18	Proposal
208	r246	chr3	160449871	TRIM59_CpG-19	Proposal
209	r247	chr3	160449877	TRIM59_CpG-20	Proposal
210	r248	chr3	160449911	TRIM59_CpG-21	Proposal
211	r249	chr3	160449937	TRIM59_CpG-22	Proposal
212	r250	chr3	160449941	TRIM59_CpG-23	Proposal
213	r251	chr3	160449951	TRIM59_CpG-24	Proposal
214	r252	chr3	160449958	TRIM59_CpG-25	Proposal
215	r253	chr3	160449961	TRIM59_CpG-26	Proposal
216	r254	chr3	160449963	TRIM59_CpG-27	Proposal
217	r255	chr3	160449968	TRIM59_CpG-28	Proposal
218	r256	chr3	160449980	TRIM59_CpG-29	Proposal
219	r257	chr3	160450001	TRIM59_CpG-30	Proposal
220	r258	chr3	160450013	TRIM59_CpG-31	Proposal
221	r259	chr3	160450015	TRIM59_CpG-32	Proposal
222	r260	chr3	160450021	TRIM59_CpG-33	Proposal
223	r261	chr3	160450030	TRIM59_CpG-34	Proposal
224	r262	chr3	160450037	TRIM59_CpG-35	Proposal
225	r263	chr3	160450044	TRIM59_CpG-36	Proposal
226	r264	chr3	160450058	TRIM59_CpG-37	Proposal
227	r265	chr3	160450066	TRIM59_CpG-38	Proposal
228	r266	chr3	160450091	TRIM59_CpG-39	Proposal
229	r267	chr3	160450105	TRIM59_CpG-40	Proposal
230	r268	chr3	160450120	TRIM59_CpG-41	Proposal
231	r269	chr3	160450133	TRIM59_CpG-42	Proposal
232	r270	chr3	160450143	TRIM59_CpG-43	Proposal
**233**	**r20**	**chr3**	**160450172**	**TRIM59_CpG-44**	**Mandatory**
**234**	**r21**	**chr3**	**160450174**	**TRIM59_CpG-45**	**Mandatory**

**235**	**r22**	**chr3**	**160450179**	**TRIM59_CpG-46**	**Mandatory**
**236**	**r23**	**chr3**	**160450184**	**TRIM59_CpG-47**	**Mandatory**
**237**	**r24**	**chr3**	**160450189**	**TRIM59_CpG-48**	**Mandatory**
**238**	**r25**	**chr3**	**160450192**	**TRIM59_CpG-49**	**Mandatory**
**239**	**r26**	**chr3**	**160450199**	**TRIM59_CpG-50**	**Mandatory**
**240**	**r27**	**chr3**	**160450202**	**TRIM59_CpG-51**	**Mandatory**
241	r271	chr3	160450231	TRIM59_CpG-52	Proposal
242	r272	chr3	160450238	TRIM59_CpG-53	Proposal
243	r273	chr3	160450243	TRIM59_CpG-54	Proposal
244	r274	chr3	160450268	TRIM59_CpG-55	Proposal
245	r275	chr3	160450283	TRIM59_CpG-56	Proposal
246	r276	chr3	160450299	TRIM59_CpG-57	Proposal
247	r277	chr3	160450307	TRIM59_CpG-58	Proposal
248	r278	chr3	160450315	TRIM59_CpG-59	Proposal
249	r279	chr3	160450348	TRIM59_CpG-60	Proposal
250	r280	chr3	160450354	TRIM59_CpG-61	Proposal
251	r281	chr3	160450361	TRIM59_CpG-62	Proposal
252	r282	chr3	160450364	TRIM59_CpG-63	Proposal
253	r283	chr3	160450388	TRIM59_CpG-64	Proposal
254	r284	chr3	160450395	TRIM59_CpG-65	Proposal
255	r285	chr3	160450411	TRIM59_CpG-66	Proposal
256	r286	chr6	11044191	ELOVL2_CpG-1	Proposal
257	r287	chr6	11044193	ELOVL2_CpG-2	Proposal
258	r288	chr6	11044196	ELOVL2_CpG-3	Proposal
259	r289	chr6	11044202	ELOVL2_CpG-4	Proposal
260	r290	chr6	11044208	ELOVL2_CpG-5	Proposal
261	r291	chr6	11044211	ELOVL2_CpG-6	Proposal
262	r292	chr6	11044213	ELOVL2_CpG-7	Proposal
263	r293	chr6	11044215	ELOVL2_CpG-8	Proposal
264	r294	chr6	11044235	ELOVL2_CpG-9	Proposal
265	r295	chr6	11044240	ELOVL2_CpG-10	Proposal
266	r296	chr6	11044249	ELOVL2_CpG-11	Proposal
267	r297	chr6	11044251	ELOVL2_CpG-12	Proposal
268	r298	chr6	11044254	ELOVL2_CpG-13	Proposal
269	r299	chr6	11044259	ELOVL2_CpG-14	Proposal
270	r300	chr6	11044263	ELOVL2_CpG-15	Proposal
271	r301	chr6	11044267	ELOVL2_CpG-16	Proposal
272	r302	chr6	11044270	ELOVL2_CpG-17	Proposal
273	r303	chr6	11044275	ELOVL2_CpG-18	Proposal
274	r304	chr6	11044291	ELOVL2_CpG-19	Proposal
275	r305	chr6	11044305	ELOVL2_CpG-20	Proposal
276	r306	chr6	11044308	ELOVL2_CpG-21	Proposal
277	r307	chr6	11044316	ELOVL2_CpG-22	Proposal
278	r308	chr6	11044319	ELOVL2_CpG-23	Proposal
279	r309	chr6	11044323	ELOVL2_CpG-24	Proposal
280	r310	chr6	11044325	ELOVL2_CpG-25	Proposal
281	r311	chr6	11044328	ELOVL2_CpG-26	Proposal
282	r312	chr6	11044350	ELOVL2_CpG-27	Proposal

283	r313	chr6	11044354	ELOVL2_CpG-28	Proposal
284	r314	chr6	11044357	ELOVL2_CpG-29	Proposal
285	r315	chr6	11044359	ELOVL2_CpG-30	Proposal
286	r316	chr6	11044361	ELOVL2_CpG-31	Proposal
287	r317	chr6	11044369	ELOVL2_CpG-32	Proposal
288	r318	chr6	11044373	ELOVL2_CpG-33	Proposal
289	r319	chr6	11044375	ELOVL2_CpG-34	Proposal
290	r320	chr6	11044383	ELOVL2_CpG-35	Proposal
291	r321	chr6	11044391	ELOVL2_CpG-36	Proposal
292	r322	chr6	11044397	ELOVL2_CpG-37	Proposal
293	r323	chr6	11044399	ELOVL2_CpG-38	Proposal
294	r324	chr6	11044402	ELOVL2_CpG-39	Proposal
295	r325	chr6	11044405	ELOVL2_CpG-40	Proposal
296	r326	chr6	11044408	ELOVL2_CpG-41	Proposal
297	r327	chr6	11044410	ELOVL2_CpG-42	Proposal
298	r328	chr6	11044412	ELOVL2_CpG-43	Proposal
299	r329	chr6	11044415	ELOVL2_CpG-44	Proposal
300	r330	chr6	11044420	ELOVL2_CpG-45	Proposal
301	r331	chr6	11044427	ELOVL2_CpG-46	Proposal
302	r332	chr6	11044433	ELOVL2_CpG-47	Proposal
303	r333	chr6	11044438	ELOVL2_CpG-48	Proposal
304	r334	chr6	11044444	ELOVL2_CpG-49	Proposal
305	r335	chr6	11044467	ELOVL2_CpG-50	Proposal
306	r336	chr6	11044470	ELOVL2_CpG-51	Proposal
307	r337	chr6	11044474	ELOVL2_CpG-52	Proposal
308	r338	chr6	11044477	ELOVL2_CpG-53	Proposal
309	r339	chr6	11044479	ELOVL2_CpG-54	Proposal
310	r340	chr6	11044490	ELOVL2_CpG-55	Proposal
311	r341	chr6	11044492	ELOVL2_CpG-56	Proposal
312	r342	chr6	11044494	ELOVL2_CpG-57	Proposal
313	r343	chr6	11044505	ELOVL2_CpG-58	Proposal
314	r344	chr6	11044521	ELOVL2_CpG-59	Proposal
315	r345	chr6	11044524	ELOVL2_CpG-60	Proposal
316	r346	chr6	11044531	ELOVL2_CpG-61	Proposal
317	r347	chr6	11044539	ELOVL2_CpG-62	Proposal
318	r348	chr6	11044545	ELOVL2_CpG-63	Proposal
319	r349	chr6	11044547	ELOVL2_CpG-64	Proposal
320	r350	chr6	11044549	ELOVL2_CpG-65	Proposal
321	r351	chr6	11044563	ELOVL2_CpG-66	Proposal
322	r352	chr6	11044567	ELOVL2_CpG-67	Proposal
323	r353	chr6	11044573	ELOVL2_CpG-68	Proposal
324	r354	chr6	11044579	ELOVL2_CpG-69	Proposal
325	r355	chr6	11044581	ELOVL2_CpG-70	Proposal
326	r356	chr6	11044585	ELOVL2_CpG-71	Proposal
327	r357	chr6	11044587	ELOVL2_CpG-72	Proposal
328	r358	chr6	11044590	ELOVL2_CpG-73	Proposal
329	r359	chr6	11044600	ELOVL2_CpG-74	Proposal
330	r360	chr6	11044604	ELOVL2_CpG-75	Proposal
331	r361	chr6	11044608	ELOVL2_CpG-76	Proposal

332	r362	chr6	11044611	ELOVL2_CpG-77	Proposal
**333**	**r28**	**chr6**	**11044628**	**ELOVL2_CpG-78**	**Desirable**
**334**	**r29**	**chr6**	**11044631**	**ELOVL2_CpG-79**	**Desirable**
**335**	**r30**	**chr6**	**11044634**	**ELOVL2_CpG-80**	**Mandatory**
**336**	**r31**	**chr6**	**11044640**	**ELOVL2_CpG-81**	**Mandatory**
**337**	**r32**	**chr6**	**11044642**	**ELOVL2_CpG-82**	**Mandatory**
**338**	**r33**	**chr6**	**11044644**	**ELOVL2_CpG-83**	**Mandatory**
**339**	**r34**	**chr6**	**11044647**	**ELOVL2_CpG-84**	**Mandatory**
**340**	**r35**	**chr6**	**11044655**	**ELOVL2_CpG-85**	**Mandatory**
**341**	**r36**	**chr6**	**11044661**	**ELOVL2_CpG-86**	**Mandatory**
342	r363	chr6	11044683	ELOVL2_CpG-87	Proposal
343	r364	chr6	11044695	ELOVL2_CpG-88	Proposal
344	r365	chr6	11044702	ELOVL2_CpG-89	Proposal
345	r366	chr6	11044710	ELOVL2_CpG-90	Proposal
346	r664	chr6	11044726	ELOVL2_CpG-91	Detected
347	r665	chr6	11044735	ELOVL2_CpG-92	Detected
348	r666	chr6	11044741	ELOVL2_CpG-93	Detected
349	r367	chr6	25652153	SCGN_CpG-1	Proposal
350	r368	chr6	25652155	SCGN_CpG-2	Proposal
351	r369	chr6	25652162	SCGN_CpG-3	Proposal
352	r370	chr6	25652167	SCGN_CpG-4	Proposal
353	r371	chr6	25652177	SCGN_CpG-5	Proposal
354	r372	chr6	25652179	SCGN_CpG-6	Proposal
355	r373	chr6	25652188	SCGN_CpG-7	Proposal
356	r374	chr6	25652193	SCGN_CpG-8	Proposal
357	r375	chr6	25652205	SCGN_CpG-9	Proposal
358	r376	chr6	25652231	SCGN_CpG-10	Proposal
359	r377	chr6	25652239	SCGN_CpG-11	Proposal
360	r378	chr6	25652242	SCGN_CpG-12	Proposal
361	r379	chr6	25652247	SCGN_CpG-13	Proposal
362	r380	chr6	25652257	SCGN_CpG-14	Proposal
363	r381	chr6	25652261	SCGN_CpG-15	Proposal
364	r382	chr6	25652263	SCGN_CpG-16	Proposal
365	r383	chr6	25652303	SCGN_CpG-17	Proposal
366	r384	chr6	25652334	SCGN_CpG-18	Proposal
**367**	**r37**	**chr6**	**25652372**	**SCGN_CpG-19**	**Mandatory**
**368**	**r38**	**chr6**	**25652374**	**SCGN_CpG-20**	**Mandatory**
**369**	**r39**	**chr6**	**25652377**	**SCGN_CpG-21**	**Mandatory**
**370**	**r40**	**chr6**	**25652391**	**SCGN_CpG-22**	**Mandatory**
**371**	**r41**	**chr6**	**25652394**	**SCGN_CpG-23**	**Mandatory**
**372**	**r42**	**chr6**	**25652416**	**SCGN_CpG-24**	**Mandatory**
**373**	**r43**	**chr6**	**25652423**	**SCGN_CpG-25**	**Mandatory**
**374**	**r44**	**chr6**	**25652434**	**SCGN_CpG-26**	**Mandatory**
**375**	**r45**	**chr6**	**25652442**	**SCGN_CpG-27**	**Mandatory**
**376**	**r46**	**chr6**	**25652445**	**SCGN_CpG-28**	**Mandatory**
377	r385	chr6	25652470	SCGN_CpG-29	Proposal
378	r386	chr6	25652480	SCGN_CpG-30	Proposal
379	r667	chr6	25652123	SCGN_CpG-31	Detected

380	r387	chr7	130733971	KLF14_CpG-1	Proposal
381	r388	chr7	130733973	KLF14_CpG-2	Proposal
382	r389	chr7	130733994	KLF14_CpG-3	Proposal
383	r390	chr7	130733997	KLF14_CpG-4	Proposal
384	r391	chr7	130734000	KLF14_CpG-5	Proposal
385	r392	chr7	130734016	KLF14_CpG-6	Proposal
386	r393	chr7	130734021	KLF14_CpG-7	Proposal
387	r394	chr7	130734024	KLF14_CpG-8	Proposal
388	r395	chr7	130734028	KLF14_CpG-9	Proposal
389	r396	chr7	130734042	KLF14_CpG-10	Proposal
390	r397	chr7	130734046	KLF14_CpG-11	Proposal
391	r398	chr7	130734050	KLF14_CpG-12	Proposal
392	r399	chr7	130734053	KLF14_CpG-13	Proposal
393	r400	chr7	130734056	KLF14_CpG-14	Proposal
394	r401	chr7	130734060	KLF14_CpG-15	Proposal
395	r402	chr7	130734063	KLF14_CpG-16	Proposal
396	r403	chr7	130734067	KLF14_CpG-17	Proposal
397	r404	chr7	130734071	KLF14_CpG-18	Proposal
398	r405	chr7	130734073	KLF14_CpG-19	Proposal
399	r406	chr7	130734077	KLF14_CpG-20	Proposal
400	r407	chr7	130734081	KLF14_CpG-21	Proposal
401	r408	chr7	130734083	KLF14_CpG-22	Proposal
402	r409	chr7	130734087	KLF14_CpG-23	Proposal
403	r410	chr7	130734090	KLF14_CpG-24	Proposal
404	r411	chr7	130734099	KLF14_CpG-25	Proposal
405	r412	chr7	130734105	KLF14_CpG-26	Proposal
406	r413	chr7	130734109	KLF14_CpG-27	Proposal
407	r414	chr7	130734114	KLF14_CpG-28	Proposal
408	r415	chr7	130734122	KLF14_CpG-29	Proposal
409	r416	chr7	130734127	KLF14_CpG-30	Proposal
410	r417	chr7	130734142	KLF14_CpG-31	Proposal
411	r418	chr7	130734150	KLF14_CpG-32	Proposal
412	r419	chr7	130734153	KLF14_CpG-33	Proposal
413	r420	chr7	130734160	KLF14_CpG-34	Proposal
414	r421	chr7	130734170	KLF14_CpG-35	Proposal
415	r422	chr7	130734173	KLF14_CpG-36	Proposal
416	r423	chr7	130734176	KLF14_CpG-37	Proposal
417	r424	chr7	130734179	KLF14_CpG-38	Proposal
418	r425	chr7	130734182	KLF14_CpG-39	Proposal
419	r426	chr7	130734188	KLF14_CpG-40	Proposal
420	r427	chr7	130734191	KLF14_CpG-41	Proposal
421	r428	chr7	130734197	KLF14_CpG-42	Proposal
422	r429	chr7	130734200	KLF14_CpG-43	Proposal
423	r430	chr7	130734203	KLF14_CpG-44	Proposal
424	r431	chr7	130734207	KLF14_CpG-45	Proposal
425	r432	chr7	130734209	KLF14_CpG-46	Proposal
426	r433	chr7	130734211	KLF14_CpG-47	Proposal
427	r434	chr7	130734218	KLF14_CpG-48	Proposal
428	r435	chr7	130734223	KLF14_CpG-49	Proposal

429	r436	chr7	130734230	KLF14_CpG-50	Proposal
430	r437	chr7	130734232	KLF14_CpG-51	Proposal
431	r438	chr7	130734241	KLF14_CpG-52	Proposal
432	r439	chr7	130734246	KLF14_CpG-53	Proposal
433	r440	chr7	130734253	KLF14_CpG-54	Proposal
434	r441	chr7	130734255	KLF14_CpG-55	Proposal
435	r442	chr7	130734261	KLF14_CpG-56	Proposal
436	r443	chr7	130734272	KLF14_CpG-57	Proposal
437	r444	chr7	130734277	KLF14_CpG-58	Proposal
438	r445	chr7	130734282	KLF14_CpG-59	Proposal
439	r446	chr7	130734297	KLF14_CpG-60	Proposal
440	r447	chr7	130734302	KLF14_CpG-61	Proposal
441	r448	chr7	130734304	KLF14_CpG-62	Proposal
442	r449	chr7	130734306	KLF14_CpG-63	Proposal
443	r450	chr7	130734313	KLF14_CpG-64	Proposal
**444**	**r47**	**chr7**	**130734355**	**KLF14_CpG-65**	**Mandatory**
**445**	**r48**	**chr7**	**130734357**	**KLF14_CpG-66**	**Mandatory**
**446**	**r49**	**chr7**	**130734372**	**KLF14_CpG-67**	**Mandatory**
**447**	**r50**	**chr7**	**130734375**	**KLF14_CpG-68**	**Mandatory**
448	r451	chr7	130734398	KLF14_CpG-69	Proposal
449	r452	chr7	130734412	KLF14_CpG-70	Proposal
450	r453	chr7	130734437	KLF14_CpG-71	Proposal
451	r454	chr7	130734450	KLF14_CpG-72	Proposal
452	r455	chr7	130734457	KLF14_CpG-73	Proposal
453	r456	chr7	130734460	KLF14_CpG-74	Proposal
454	r457	chr7	130734464	KLF14_CpG-75	Proposal
455	r458	chr7	130734472	KLF14_CpG-76	Proposal
456	r459	chr7	130734475	KLF14_CpG-77	Proposal
457	r460	chr7	130734491	KLF14_CpG-78	Proposal
458	r461	chr7	130734493	KLF14_CpG-79	Proposal
459	r668	chr7	130733883	KLF14_CpG-80	Detected
460	r669	chr7	130733890	KLF14_CpG-81	Detected
461	r670	chr7	130733893	KLF14_CpG-82	Detected
462	r671	chr7	130733896	KLF14_CpG-83	Detected
463	r672	chr7	130733899	KLF14_CpG-84	Detected
464	r673	chr7	130733911	KLF14_CpG-85	Detected
465	r674	chr7	130733919	KLF14_CpG-86	Detected
466	r675	chr7	130733929	KLF14_CpG-87	Detected
467	r676	chr7	130733931	KLF14_CpG-88	Detected
468	r677	chr7	130733937	KLF14_CpG-89	Detected
469	r678	chr7	130733944	KLF14_CpG-90	Detected
470	r679	chr7	130733947	KLF14_CpG-91	Detected
471	r680	chr7	130733951	KLF14_CpG-92	Detected
472	r681	chr7	130733954	KLF14_CpG-93	Detected
473	r682	chr7	130733964	KLF14_CpG-94	Detected
474	r683	chr7	130733967	KLF14_CpG-95	Detected
475	r684	chr7	130734556	KLF14_CpG-96	Detected

476	r685	chr7	130734569	KLF14_CpG-97	Detected
477	r686	chr7	130734579	KLF14_CpG-98	Detected
478	r462	chr16	49857692	ZNF423_CpG-1	Proposal
479	r463	chr16	49857694	ZNF423_CpG-2	Proposal
480	r464	chr16	49857718	ZNF423_CpG-3	Proposal
481	r465	chr16	49857771	ZNF423_CpG-4	Proposal
482	r466	chr16	49857773	ZNF423_CpG-5	Proposal
483	r467	chr16	49857812	ZNF423_CpG-6	Proposal
484	r468	chr16	49857828	ZNF423_CpG-7	Proposal
485	r469	chr16	49857838	ZNF423_CpG-8	Proposal
**486**	**r51**	**chr16**	**49857841**	**ZNF423_CpG-9**	**Mandatory**
**487**	**r52**	**chr16**	**49857843**	**ZNF423_CpG-10**	**Mandatory**
**488**	**r53**	**chr16**	**49857845**	**ZNF423_CpG-11**	**Mandatory**
489	r470	chr16	49857852	ZNF423_CpG-12	Proposal
490	r471	chr16	49857866	ZNF423_CpG-13	Proposal
491	r472	chr16	49857885	ZNF423_CpG-14	Proposal
492	r473	chr16	49857895	ZNF423_CpG-15	Proposal
493	r474	chr16	49857932	ZNF423_CpG-16	Proposal
494	r475	chr16	49857946	ZNF423_CpG-17	Proposal
495	r476	chr16	49857953	ZNF423_CpG-18	Proposal
496	r477	chr16	49857984	ZNF423_CpG-19	Proposal
497	r478	chr16	49857995	ZNF423_CpG-20	Proposal
498	r479	chr16	49858006	ZNF423_CpG-21	Proposal
499	r480	chr16	49858012	ZNF423_CpG-22	Proposal
500	r481	chr16	49858018	ZNF423_CpG-23	Proposal
501	r482	chr16	49858029	ZNF423_CpG-24	Proposal
502	r483	chr16	49858040	ZNF423_CpG-25	Proposal
503	r484	chr16	49858053	ZNF423_CpG-26	Proposal
504	r485	chr16	49858056	ZNF423_CpG-27	Proposal
505	r486	chr16	49858058	ZNF423_CpG-28	Proposal
506	r487	chr16	49858063	ZNF423_CpG-29	Proposal
507	r488	chr16	49858077	ZNF423_CpG-30	Proposal
508	r489	chr16	49858082	ZNF423_CpG-31	Proposal
509	r490	chr16	49858090	ZNF423_CpG-32	Proposal
510	r491	chr16	49858093	ZNF423_CpG-33	Proposal
511	r492	chr16	49858095	ZNF423_CpG-34	Proposal
512	r493	chr16	49858098	ZNF423_CpG-35	Proposal
513	r494	chr16	49858119	ZNF423_CpG-36	Proposal
514	r495	chr16	49858124	ZNF423_CpG-37	Proposal
515	r496	chr16	49858156	ZNF423_CpG-38	Proposal
516	r497	chr16	49858164	ZNF423_CpG-39	Proposal
517	r498	chr16	49858170	ZNF423_CpG-40	Proposal
518	r499	chr16	49858194	ZNF423_CpG-41	Proposal
519	r500	chr16	49858204	ZNF423_CpG-42	Proposal
520	r501	chr16	49858211	ZNF423_CpG-43	Proposal
521	r502	chr16	49858214	ZNF423_CpG-44	Proposal
522	r687	chr16	49857593	ZNF423_CpG-45	Detected
523	r688	chr16	49857597	ZNF423_CpG-46	Detected

524	r689	chr16	49857658	ZNF423_CpG-47	Detected
525	r690	chr16	49857662	ZNF423_CpG-48	Detected
526	r691	chr16	49858223	ZNF423_CpG-49	Detected
527	r692	chr16	49858226	ZNF423_CpG-50	Detected
528	r693	chr16	49858232	ZNF423_CpG-51	Detected
529	r694	chr16	49858236	ZNF423_CpG-52	Detected
530	r695	chr16	49858241	ZNF423_CpG-53	Detected
531	r696	chr16	49858243	ZNF423_CpG-54	Detected
532	r697	chr16	49858246	ZNF423_CpG-55	Detected
533	r698	chr16	49858250	ZNF423_CpG-56	Detected
534	r699	chr16	49858257	ZNF423_CpG-57	Detected
535	r700	chr16	49858268	ZNF423_CpG-58	Detected
536	r701	chr16	49858270	ZNF423_CpG-59	Detected
537	r702	chr16	49858275	ZNF423_CpG-60	Detected
538	r703	chr16	49858278	ZNF423_CpG-61	Detected
539	r704	chr16	49858293	ZNF423_CpG-62	Detected
540	r705	chr16	49858301	ZNF423_CpG-63	Detected
**541**	**r54**	**chr17**	**3476237**	**ASPA_CpG-1**	**Mandatory**
**542**	**r55**	**chr17**	**3476273**	**ASPA_CpG-2**	**Mandatory**
543	r503	chr17	3471783	ASPA_CpG-3	Proposal
544	r504	chr17	3471786	ASPA_CpG-4	Proposal
545	r505	chr17	3471795	ASPA_CpG-5	Proposal
546	r506	chr17	3471799	ASPA_CpG-6	Proposal
547	r507	chr17	3471801	ASPA_CpG-7	Proposal
548	r508	chr17	3471810	ASPA_CpG-8	Proposal
549	r509	chr17	3471812	ASPA_CpG-9	Proposal
550	r510	chr17	3471828	ASPA_CpG-10	Proposal
551	r511	chr17	3471834	ASPA_CpG-11	Proposal
552	r512	chr17	3471837	ASPA_CpG-12	Proposal
553	r513	chr17	3471844	ASPA_CpG-13	Proposal
554	r514	chr17	3471846	ASPA_CpG-14	Proposal
555	r515	chr17	3471852	ASPA_CpG-15	Proposal
556	r516	chr17	3471861	ASPA_CpG-16	Proposal
557	r517	chr17	3471869	ASPA_CpG-17	Proposal
558	r518	chr17	3471872	ASPA_CpG-18	Proposal
559	r519	chr17	3471874	ASPA_CpG-19	Proposal
560	r520	chr17	3471886	ASPA_CpG-20	Proposal
561	r521	chr17	3471901	ASPA_CpG-21	Proposal
562	r522	chr17	3471911	ASPA_CpG-22	Proposal
563	r523	chr17	3471915	ASPA_CpG-23	Proposal
564	r524	chr17	3471917	ASPA_CpG-24	Proposal
565	r525	chr17	3471923	ASPA_CpG-25	Proposal
566	r526	chr17	3471929	ASPA_CpG-26	Proposal
567	r527	chr17	3471942	ASPA_CpG-27	Proposal
568	r528	chr17	3472212	ASPA_CpG-28	Proposal
569	r529	chr17	3472240	ASPA_CpG-29	Proposal
570	r530	chr17	3472280	ASPA_CpG-30	Proposal
571	r531	chr17	3472293	ASPA_CpG-31	Proposal

572	r532	chr17	3472301	ASPA_CpG-32	Proposal
573	r533	chr17	3472307	ASPA_CpG-33	Proposal
574	r534	chr17	3472315	ASPA_CpG-34	Proposal
575	r535	chr17	3472318	ASPA_CpG-35	Proposal
576	r536	chr17	3472326	ASPA_CpG-36	Proposal
577	r537	chr17	3472336	ASPA_CpG-37	Proposal
578	r538	chr17	3472347	ASPA_CpG-38	Proposal
579	r539	chr17	3472351	ASPA_CpG-39	Proposal
580	r540	chr17	3472358	ASPA_CpG-40	Proposal
581	r541	chr17	3472366	ASPA_CpG-41	Proposal
582	r542	chr17	3472383	ASPA_CpG-42	Proposal
583	r543	chr17	3472385	ASPA_CpG-43	Proposal
584	r544	chr17	3472394	ASPA_CpG-44	Proposal
585	r545	chr17	3472404	ASPA_CpG-45	Proposal
586	r706	chr17	3471773	ASPA_CpG-46	Detected
587	r707	chr17	3472175	ASPA_CpG-47	Detected
588	r708	chr17	3472186	ASPA_CpG-48	Detected
589	r546	chr17	44385472	ITGA2B_CpG-1	Proposal
590	r547	chr17	44385475	ITGA2B_CpG-2	Proposal
591	r548	chr17	44385484	ITGA2B_CpG-3	Proposal
592	r549	chr17	44385491	ITGA2B_CpG-4	Proposal
593	r550	chr17	44385500	ITGA2B_CpG-5	Proposal
594	r551	chr17	44385511	ITGA2B_CpG-6	Proposal
595	r552	chr17	44385514	ITGA2B_CpG-7	Proposal
596	r553	chr17	44385516	ITGA2B_CpG-8	Proposal
597	r554	chr17	44385524	ITGA2B_CpG-9	Proposal
598	r555	chr17	44385535	ITGA2B_CpG-10	Proposal
599	r556	chr17	44385544	ITGA2B_CpG-11	Proposal
600	r557	chr17	44385566	ITGA2B_CpG-12	Proposal
601	r558	chr17	44385574	ITGA2B_CpG-13	Proposal
602	r559	chr17	44385590	ITGA2B_CpG-14	Proposal
**603**	**r56**	**chr17**	**44385592**	**ITGA2B_CpG-15**	**Mandatory**
604	r560	chr17	44385608	ITGA2B_CpG-16	Proposal
605	r561	chr17	44385611	ITGA2B_CpG-17	Proposal
606	r562	chr17	44385613	ITGA2B_CpG-18	Proposal
607	r563	chr17	44385616	ITGA2B_CpG-19	Proposal
**608**	**r57**	**chr17**	**44385620**	**ITGA2B_CpG-20**	**Mandatory**
**609**	**r58**	**chr17**	**44385653**	**ITGA2B_CpG-21**	**Mandatory**
610	r564	chr17	44385657	ITGA2B_CpG-22	Proposal
611	r565	chr17	44385689	ITGA2B_CpG-23	Proposal
612	r566	chr17	44385710	ITGA2B_CpG-24	Proposal
613	r567	chr17	44385743	ITGA2B_CpG-25	Proposal
614	r568	chr17	44385747	ITGA2B_CpG-26	Proposal
615	r569	chr17	44385749	ITGA2B_CpG-27	Proposal
616	r570	chr17	44385789	ITGA2B_CpG-28	Proposal
617	r571	chr17	44385812	ITGA2B_CpG-29	Proposal
618	r572	chr17	44385814	ITGA2B_CpG-30	Proposal
619	r573	chr17	44385832	ITGA2B_CpG-31	Proposal

620	r574	chr17	44385835	ITGA2B_CpG-32	Proposal
621	r575	chr17	44385847	ITGA2B_CpG-33	Proposal
622	r576	chr17	44385851	ITGA2B_CpG-34	Proposal
623	r577	chr17	44385854	ITGA2B_CpG-35	Proposal
624	r578	chr17	44385870	ITGA2B_CpG-36	Proposal
625	r579	chr17	44385909	ITGA2B_CpG-37	Proposal
626	r580	chr17	44385947	ITGA2B_CpG-38	Proposal
627	r581	chr17	44385957	ITGA2B_CpG-39	Proposal
628	r582	chr17	44385967	ITGA2B_CpG-40	Proposal
629	r612	chr18	68722125	CCDC102B_CpG-1	Proposal
630	r613	chr18	68722149	CCDC102B_CpG-2	Proposal
**631**	**r59**	**chr18**	**68722183**	**CCDC102B_CpG-3**	**Mandatory**
**632**	**r60**	**chr18**	**68722210**	**CCDC102B_CpG-4**	**Mandatory**
633	r583	chr18	68715042	CCDC102B_CpG-5	Proposal
634	r584	chr18	68715045	CCDC102B_CpG-6	Proposal
635	r585	chr18	68715053	CCDC102B_CpG-7	Proposal
636	r586	chr18	68715057	CCDC102B_CpG-8	Proposal
637	r587	chr18	68715069	CCDC102B_CpG-9	Proposal
638	r588	chr18	68715075	CCDC102B_CpG-10	Proposal
639	r589	chr18	68715080	CCDC102B_CpG-11	Proposal
640	r590	chr18	68715085	CCDC102B_CpG-12	Proposal
641	r591	chr18	68715099	CCDC102B_CpG-13	Proposal
642	r592	chr18	68715101	CCDC102B_CpG-14	Proposal
643	r593	chr18	68715110	CCDC102B_CpG-15	Proposal
644	r594	chr18	68715116	CCDC102B_CpG-16	Proposal
645	r595	chr18	68715126	CCDC102B_CpG-17	Proposal
646	r596	chr18	68715137	CCDC102B_CpG-18	Proposal
647	r597	chr18	68715143	CCDC102B_CpG-19	Proposal
648	r598	chr18	68715151	CCDC102B_CpG-20	Proposal
649	r599	chr18	68715153	CCDC102B_CpG-21	Proposal
650	r600	chr18	68715161	CCDC102B_CpG-22	Proposal
651	r601	chr18	68715164	CCDC102B_CpG-23	Proposal
652	r602	chr18	68715170	CCDC102B_CpG-24	Proposal
653	r603	chr18	68715181	CCDC102B_CpG-25	Proposal
654	r604	chr18	68715184	CCDC102B_CpG-26	Proposal
655	r605	chr18	68715190	CCDC102B_CpG-27	Proposal
656	r606	chr18	68715196	CCDC102B_CpG-28	Proposal
657	r607	chr18	68715202	CCDC102B_CpG-29	Proposal
658	r608	chr18	68715228	CCDC102B_CpG-30	Proposal
659	r609	chr18	68715234	CCDC102B_CpG-31	Proposal
660	r610	chr18	68715249	CCDC102B_CpG-32	Proposal
661	r611	chr18	68715259	CCDC102B_CpG-33	Proposal
662	r709	chr18	68715295	CCDC102B_CpG-34	Detected
663	r614	chr19	18232856	PDE4C_CpG-1	Proposal
664	r615	chr19	18232891	PDE4C_CpG-2	Proposal
665	r616	chr19	18232901	PDE4C_CpG-3	Proposal
666	r617	chr19	18232903	PDE4C_CpG-4	Proposal
667	r618	chr19	18232905	PDE4C_CpG-5	Proposal

668	r619	chr19	18232916	PDE4C_CpG-6	Proposal
669	r620	chr19	18232918	PDE4C_CpG-7	Proposal
670	r621	chr19	18232939	PDE4C_CpG-8	Proposal
671	r622	chr19	18232950	PDE4C_CpG-9	Proposal
672	r623	chr19	18232966	PDE4C_CpG-10	Proposal
673	r624	chr19	18232980	PDE4C_CpG-11	Proposal
674	r625	chr19	18232982	PDE4C_CpG-12	Proposal
675	r626	chr19	18232997	PDE4C_CpG-13	Proposal
676	r627	chr19	18233001	PDE4C_CpG-14	Proposal
677	r628	chr19	18233003	PDE4C_CpG-15	Proposal
678	r629	chr19	18233020	PDE4C_CpG-16	Proposal
679	r630	chr19	18233022	PDE4C_CpG-17	Proposal
680	r631	chr19	18233030	PDE4C_CpG-18	Proposal
681	r632	chr19	18233032	PDE4C_CpG-19	Proposal
**682**	**r61**	**chr19**	**18233042**	**PDE4C_CpG-20**	**Mandatory**
**683**	**r62**	**chr19**	**18233048**	**PDE4C_CpG-21**	**Mandatory**
**684**	**r63**	**chr19**	**18233058**	**PDE4C_CpG-22**	**Mandatory**
**685**	**r64**	**chr19**	**18233070**	**PDE4C_CpG-23**	**Mandatory**
**686**	**r65**	**chr19**	**18233079**	**PDE4C_CpG-24**	**Mandatory**
**687**	**r66**	**chr19**	**18233082**	**PDE4C_CpG-25**	**Mandatory**
**688**	**r67**	**chr19**	**18233091**	**PDE4C_CpG-26**	**Mandatory**
**689**	**r68**	**chr19**	**18233105**	**PDE4C_CpG-27**	**Mandatory**
**690**	**r69**	**chr19**	**18233127**	**PDE4C_CpG-28**	**Desirable**
**691**	**r70**	**chr19**	**18233131**	**PDE4C_CpG-29**	**Desirable**
**692**	**r71**	**chr19**	**18233133**	**PDE4C_CpG-30**	**Desirable**
693	r633	chr19	18233139	PDE4C_CpG-31	Proposal
694	r634	chr19	18233170	PDE4C_CpG-32	Proposal
695	r635	chr19	18233173	PDE4C_CpG-33	Proposal
696	r636	chr19	18233193	PDE4C_CpG-34	Proposal
697	r637	chr19	18233232	PDE4C_CpG-35	Proposal
698	r638	chr19	18233238	PDE4C_CpG-36	Proposal
699	r639	chr19	18233244	PDE4C_CpG-37	Proposal
700	r640	chr19	18233248	PDE4C_CpG-38	Proposal
701	r641	chr19	18233251	PDE4C_CpG-39	Proposal
702	r642	chr19	18233258	PDE4C_CpG-40	Proposal
703	r643	chr19	18233268	PDE4C_CpG-41	Proposal
704	r644	chr19	18233274	PDE4C_CpG-42	Proposal
705	r645	chr19	18233285	PDE4C_CpG-43	Proposal
706	r646	chr19	18233297	PDE4C_CpG-44	Proposal
707	r647	chr19	18233303	PDE4C_CpG-45	Proposal
708	r648	chr19	18233315	PDE4C_CpG-46	Proposal
709	r649	chr19	18233317	PDE4C_CpG-47	Proposal
710	r710	chr19	18232800	PDE4C_CpG-48	Detected
711	r711	chr19	18232826	PDE4C_CpG-49	Detected
712	r712	chr19	18233320	PDE4C_CpG-50	Detected
713	r713	chr19	18233334	PDE4C_CpG-51	Detected

Uncovered CpG sites

2. Amplicon Listing DNA Methylation Panel WG00651_MET_V2

Ítem Amplicon	Amplicon_ ID	Gene Name	Chr	Amplicon_ Start	Amplicon_ Stop	Size pb	Insert _Start	Insert _Stop	Pool _ID
1	C_SP_10.1488022	**FHL2**	chr2	105398874	105399047	173	105398900	105399020	1
2	C_SP_10.2187030	**FHL2**	chr2	105399539	105399711	172	105399571	105399688	1
3	C_SP_11.1204703	**TRIM59**	chr3	160449877	160450051	174	160449906	160450028	1
4	C_SP_11.1768899	**TRIM59**	chr3	160450285	160450456	171	160450311	160450420	1
5	C_SP_11.194094	**SCGN**	chr6	25652269	25652433	164	25652301	25652401	1
6	C_SP_11.685200	**TRIM59**	chr3	160449652	160449826	174	160449685	160449800	1
7	C_SP_1.199018	**C1ORF132**	chr1	207823588	207823750	162	207823620	207823716	1
8	C_SP_12.15750	**KLF14**	chr7	130734308	130734442	134	130734340	130734413	1
9	C_SP_12.82703	**ELOVL2**	chr6	11044150	11044305	155	11044180	11044276	1
10	C_SP_13.168400	**SCGN**	chr6	25652093	25652224	131	25652124	25652190	1
11	C_SP_14.137361	**KLF14**	chr7	130733904	130734053	149	130733927	130734030	1
12	C_SP_14.148550	**KLF14**	chr7	130734462	130734609	147	130734490	130734582	1
13	C_SP_1.601134	**C1ORF132**	chr1	207823766	207823890	124	207823792	207823857	1
14	C_SP_1.855441	**C1ORF132**	chr1	207823969	207824133	164	207824002	207824099	1
15	C_SP_2.21921	**EDARADD**	chr1	236394294	236394458	164	236394328	236394426	1
16	C_SP_3.473022	**EDARADD**	chr1	236395680	236395854	174	236395703	236395831	1
17	C_SP_3.52862	**EDARADD**	chr1	236395495	236395654	159	236395521	236395628	1
18	C_SP_3.59987	**ZNF423**	chr16	49857731	49857885	154	49857763	49857853	1
19	C_SP_3.782116	**EDARADD**	chr1	236395180	236395346	166	236395210	236395323	1
20	C_SP_4.1272394	**ZNF423**	chr16	49858158	49858327	169	49858189	49858302	1
21	C_SP_4.369	**ASPA**	chr17	3476205	3476330	125	3476243	3476294	1
22	C_SP_4.855256	**ZNF423**	chr16	49857964	49858138	174	49857988	49858102	1
23	C_SP_5.1432979	**ASPA**	chr17	3471751	3471917	166	3471774	3471894	1
24	C_SP_5.53263	**ASPA**	chr17	3472242	3472404	162	3472275	3472371	1
25	C_SP_5.604944	**ITGA2B**	chr17	44385556	44385699	143	44385591	44385666	1
26	C_SP_6.48513	**CCDC102B**	chr18	68722071	68722242	171	68722107	68722204	1
27	C_SP_6.671295	**ITGA2B**	chr17	44385776	44385930	154	44385805	44385898	1
28	C_SP_7.174597	**CCDC102B**	chr18	68715195	68715337	142	68715228	68715302	1
29	C_SP_7.359928	**CCDC102B**	chr18	68714983	68715152	169	68715018	68715127	1
30	C_SP_7.55667	**PDE4C**	chr19	18233005	18233170	165	18233031	18233142	1
31	C_SP_8.276368	**FHL2**	chr2	105399238	105399394	156	105399271	105399361	1
32	C_SP_9.205983	**TRIM59**	chr3	160450122	160450291	169	160450156	160450258	1
33	W_SP_10.105146	**ELOVL2**	chr6	11044511	11044685	174	11044539	11044647	1
34	W_SP_10.1150890	**FHL2**	chr2	105399073	105399243	170	105399111	105399212	1
35	W_SP_10.278005	**FHL2**	chr2	105398895	105399069	174	105398922	105399044	1
36	W_SP_10.538857	**FHL2**	chr2	105399611	105399756	145	105399647	105399723	1
37	W_SP_10.620524	**FHL2**	chr2	105398769	105398902	133	105398805	105398875	1
38	W_SP_10.90084	**FHL2**	chr2	105399451	105399618	167	105399482	105399582	1
39	W_SP_11.1317956	**TRIM59**	chr3	160449900	160450073	173	160449932	160450045	1
40	W_SP_11.260752	**SCGN**	chr6	25652335	25652503	168	25652367	25652471	1
41	W_SP_11.576121	**TRIM59**	chr3	160449720	160449855	135	160449751	160449832	1
42	W_SP_12.294330	**ELOVL2**	chr6	11044159	11044306	147	11044183	11044279	1
43	W_SP_12.75669	**KLF14**	chr7	130734305	130734435	130	130734341	130734401	1
44	W_SP_1.41844	**C1ORF132**	chr1	207823621	207823745	124	207823652	207823712	1

45	W_SP_14.203495	**KLF14**	chr7	130734130	130734296	166	130734153	130734264	1
46	W_SP_14.314681	**KLF14**	chr7	130733965	130734137	172	130733994	130734110	1
47	W_SP_1.50225	**C1ORF132**	chr1	207823862	207824029	167	207823895	207823999	1
48	W_SP_2.8223	**EDARADD**	chr1	236394326	236394483	157	236394359	236394452	1
49	W_SP_3.21621	**ZNF423**	chr16	49857804	49857931	127	49857837	49857898	1
50	W_SP_3.465673	**EDARADD**	chr1	236395402	236395565	163	236395432	236395530	1
51	W_SP_3.574451	**EDARADD**	chr1	236395217	236395391	174	236395245	236395368	1
52	W_SP_3.866106	**EDARADD**	chr1	236395564	236395736	172	236395592	236395712	1
53	W_SP_4.11365	**ASPA**	chr17	3476192	3476318	126	3476225	3476283	1
54	W_SP_4.44297	**ZNF423**	chr16	49857558	49857732	174	49857589	49857699	1
55	W_SP_4.650733	**ZNF423**	chr16	49858132	49858306	174	49858156	49858278	1
56	W_SP_4.652011	**ZNF423**	chr16	49857969	49858131	162	49858001	49858099	1
57	W_SP_5.117150	**ITGA2B**	chr17	44385525	44385659	134	44385551	44385626	1
58	W_SP_5.240952	**ASPA**	chr17	3472172	3472346	174	3472203	3472322	1
59	W_SP_5.292089	**ASPA**	chr17	3471801	3471968	167	3471826	3471936	1
60	W_SP_6.104393	**CCDC102B**	chr18	68722103	68722249	146	68722137	68722216	1
61	W_SP_6.776912	**ITGA2B**	chr17	44385773	44385946	173	44385805	44385918	1
62	W_SP_7.328942	**PDE4C**	chr19	18233059	18233226	167	18233082	18233195	1
63	W_SP_7.389013	**CCDC102B**	chr18	68715165	68715303	138	68715188	68715270	1
64	W_SP_7.497219	**CCDC102B**	chr18	68715000	68715148	148	68715033	68715116	1
65	W_SP_8.365338	**FHL2**	chr2	105399243	105399397	154	105399277	105399365	1
66	W_SP_9.101501	**TRIM59**	chr3	160450127	160450286	159	160450155	160450253	1
67	W_SP_9.88294	**PDE4C**	chr19	18232823	18232979	156	18232859	18232952	1
68	C_SP_10.1051761	**FHL2**	chr2	105398781	105398942	161	105398806	105398913	2
69	C_SP_10.1516	**FHL2**	chr2	105399430	105399604	174	105399466	105399581	2
70	C_SP_10.3166	**ELOVL2**	chr6	11044602	11044762	160	11044629	11044728	2
71	C_SP_10.478834	**FHL2**	chr2	105398954	105399128	174	105398984	105399095	2
72	C_SP_11.102399	**SCGN**	chr6	25652381	25652531	150	25652412	25652498	2
73	C_SP_11.686280	**TRIM59**	chr3	160449771	160449945	174	160449798	160449909	2
74	C_SP_1.21836	**C1ORF132**	chr1	207823628	207823759	131	207823661	207823726	2
75	C_SP_12.197518	**ELOVL2**	chr6	11044439	11044579	140	11044462	11044556	2
76	C_SP_12.24064	**ELOVL2**	chr6	11044217	11044352	135	11044250	11044329	2
77	C_SP_13.154518	**SCGN**	chr6	25652130	25652304	174	25652160	25652269	2
78	C_SP_14.203288	**KLF14**	chr7	130734357	130734526	169	130734389	130734489	2
79	C_SP_14.82670	**KLF14**	chr7	130733996	130734169	173	130734022	130734146	2
80	C_SP_1.781911	**C1ORF132**	chr1	207823865	207824039	174	207823895	207824012	2
81	C_SP_2.150099	**EDARADD**	chr1	236394311	236394483	172	236394344	236394448	2
82	C_SP_3.156386	**EDARADD**	chr1	236395087	236395248	161	236395120	236395220	2
83	C_SP_3.20424	**EDARADD**	chr1	236395581	236395748	167	236395605	236395718	2
84	C_SP_3.756120	**EDARADD**	chr1	236395285	236395456	171	236395312	236395419	2
85	C_SP_3.91926	**ZNF423**	chr16	49857794	49857938	144	49857827	49857904	2
86	C_SP_4.550250	**ZNF423**	chr16	49857622	49857760	138	49857652	49857729	2
87	C_SP_4.830031	**ZNF423**	chr16	49858024	49858198	174	49858053	49858171	2
88	C_SP_5.234236	**ITGA2B**	chr17	44385615	44385787	172	44385648	44385756	2
89	C_SP_5.536173	**ASPA**	chr17	3471871	3471999	128	3471898	3471967	2
90	C_SP_5.604217	**ASPA**	chr17	3472143	3472291	148	3472176	3472257	2
91	C_SP_5.95939	**ASPA**	chr17	3472328	3472493	165	3472359	3472461	2
92	C_SP_6.155496	**ITGA2B**	chr17	44385435	44385608	173	44385466	44385576	2
93	C_SP_6.30121	**CCDC102B**	chr18	68722164	68722313	149	68722197	68722280	2

94	C_SP_6.581405	**ITGA2B**	chr17	44385853	44386006	153	44385881	44385975	2
95	C_SP_7.34454	**PDE4C**	chr19	18232968	18233121	153	18232998	18233088	2
96	C_SP_7.364796	**CCDC102B**	chr18	68715103	68715277	174	68715131	68715247	2
97	C_SP_8.83786	**FHL2**	chr2	105399220	105399380	160	105399253	105399350	2
98	C_SP_9.239409	**PDE4C**	chr19	18232793	18232948	155	18232825	18232920	2
99	C_SP_9.340683	**TRIM59**	chr3	160450131	160450299	168	160450166	160450269	2
100	C_SP_9.54559	**PDE4C**	chr19	18233211	18233382	171	18233245	18233348	2
101	W_SP_10.1401301	**FHL2**	chr2	105398795	105398968	173	105398831	105398944	2
102	W_SP_10.495436	**FHL2**	chr2	105399364	105399488	124	105399396	105399458	2
103	W_SP_10.573011	**FHL2**	chr2	105398970	105399135	165	105398993	105399108	2
104	W_SP_10.579053	**FHL2**	chr2	105399481	105399655	174	105399514	105399619	2
105	W_SP_10.604241	**FHL2**	chr2	105399207	105399371	164	105399239	105399341	2
106	W_SP_10.99975	**ELOVL2**	chr6	11044601	11044774	173	11044624	11044741	2
107	W_SP_11.430269	**TRIM59**	chr3	160449799	160449945	146	160449829	160449917	2
108	W_SP_11.55571	**TRIM59**	chr3	160449645	160449792	147	160449679	160449763	2
109	W_SP_11.559264	**TRIM59**	chr3	160449981	160450155	174	160450013	160450129	2
110	W_SP_11.62890	**SCGN**	chr6	25652316	25652484	168	25652351	25652455	2
111	W_SP_11.693030	**TRIM59**	chr3	160450269	160450443	174	160450305	160450417	2
112	W_SP_13.508608	**SCGN**	chr6	25652069	25652238	169	25652102	25652211	2
113	W_SP_14.283687	**KLF14**	chr7	130734247	130734397	150	130734270	130734364	2
114	W_SP_14.332202	**KLF14**	chr7	130733854	130734014	160	130733883	130733988	2
115	W_SP_14.416896	**KLF14**	chr7	130734017	130734154	137	130734042	130734131	2
116	W_SP_14.706609	**KLF14**	chr7	130734404	130734578	174	130734437	130734540	2
117	W_SP_1.86134	**C1ORF132**	chr1	207823677	207823851	174	207823711	207823821	2
118	W_SP_2.5002	**EDARADD**	chr1	236394341	236394495	154	236394373	236394465	2
119	W_SP_3.1796339	**EDARADD**	chr1	236395647	236395810	163	236395678	236395777	2
120	W_SP_3.640975	**EDARADD**	chr1	236395486	236395643	157	236395510	236395608	2
121	W_SP_3.668698	**EDARADD**	chr1	236395306	236395439	133	236395339	236395407	2
122	W_SP_3.692421	**EDARADD**	chr1	236395136	236395310	174	236395164	236395282	2
123	W_SP_4.231860	**ZNF423**	chr16	49858065	49858209	144	49858089	49858172	2
124	W_SP_4.60927	**ASPA**	chr17	3476203	3476362	159	3476235	3476327	2
125	W_SP_4.814095	**ZNF423**	chr16	49857876	49858050	174	49857901	49858023	2
126	W_SP_4.826665	**ZNF423**	chr16	49857666	49857836	170	49857704	49857806	2
127	W_SP_5.1006701	**ASPA**	chr17	3472284	3472449	165	3472310	3472416	2
128	W_SP_5.141030	**ITGA2B**	chr17	44385575	44385740	165	44385601	44385710	2
129	W_SP_5.221167	**ASPA**	chr17	3471876	3472022	146	3471909	3471992	2
130	W_SP_6.527123	**ITGA2B**	chr17	44385871	44386024	153	44385902	44385992	2
131	W_SP_6.59846	**CCDC102B**	chr18	68722126	68722267	141	68722158	68722236	2
132	W_SP_7.249009	**CCDC102B**	chr18	68715071	68715231	160	68715099	68715196	2
133	W_SP_7.540185	**PDE4C**	chr19	18232952	18233114	162	18232980	18233088	2
134	W_SP_9.144824	**PDE4C**	chr19	18232720	18232894	174	18232757	18232858	2
135	W_SP_9.46813	**PDE4C**	chr19	18233197	18233354	157	18233226	18233326	2

3. Amplicons per Gene

(based on table 2)

GEN	Chr	Ítem Amplicon POOL 1	Ítem Amplicon POOL 2
**C1orf132**	1	7, 13, 14, 44,47	74, 80, 117
**EDARADD**	1	15, 16, 17, 19, 48, 50, 51, 52	81, 82, 83, 84, 118, 119, 120, 121, 122
**FHL2**	2	1, 2, 31, 34, 35, 36, 37, 38, 65	68, 69, 71, 97, 101, 102, 103, 104, 105
**TRIM59**	3	3, 4, 6, 32, 39, 41, 66	73, 99, 107, 108, 109, 111
**ELOVL2**	6	9, 33, 42	70, 75, 76, 106
**SCGN**	6	5, 10, 40	72, 77, 110, 112
**KLF14**	7	8, 11, 12, 43, 45, 46	78, 79, 113, 114, 115, 116
**ZNF423**	16	18, 20, 22, 49, 54, 55, 56	85, 86, 87, 123, 125, 126
**ASPA**	17	21, 23, 24, 53, 58, 59	89, 90, 91, 124, 127, 129
**ITGA2B**	17	25, 27, 57, 61	88, 92, 94, 128, 139
**CCDC102B**	18	26, 28, 29, 60, 63, 64	93, 96, 131, 132
**PDE4C**	19	30, 62, 67	95, 98, 100, 133, 134, 135
