# Supplementary material for: Development of a Model for Predicting Chronological Age Based on DNA Methylation Data from Buccal Swabs Using Ion Torrent S5 Technology
Source: Int J Mol Sci. 2026 Aug 6;27(15):7062. doi: 10.3390/ijms27157062 (PMC13466995; doi:10.3390/ijms27157062)
Supplement: Supplementary file 1 [file ijms-27-07062-s001.zip › SM3_Results_Development_age_prediction_model_FINAL_23-07-2026.pdf]

Supplementary Material S3

Results: Development of the age prediction model

Representative CpG Panel

Table 1. Final representative CpG panel per matrix with natural spline allocation.

Matrix	Strand	N_representative	N_splines	CpGs_spline
dat_W	Watson	5	2	C1orf132_CpG-5; KLF14_CpG-65
dat_C	Crick	6	1	C1orf132_CpG-12
dat_WC	Watson+Crick collapsed	7	3	C1orf132_CpG-10; ELOVL2_CpG-72; PDE4C_CpG-41

Nested Cross-Validation

Table 2. Performance obtained using nested cross-validation (5-outer × 5-inner folds).

Matrix	Strand	n_outer	n_cpg_medium	MAE	RMSE	R^2
dat_C	Crick	5	6	5.299	6.648	0.809
dat_W	Watson	5	5	4.695	5.593	0.870
dat_WC	Watson+Crick collapsed	5	7	5.729	6.869	0.789

CpG Selection Stability

Table 3. CpG selection frequency across nested-CV outer folds (≥60%)

Matrix	Strand	CpG	Gene	Folds_selected	Frequency
dat_C	Crick	C1orf132_CpG-12	C1orf132	5	1.0
dat_C	Crick	EDARADD_CpG-2	EDARADD	5	1.0
dat_C	Crick	TRIM59_CpG-65	TRIM59	5	1.0
dat_C	Crick	KLF14_CpG-94	KLF14	4	0.8
dat_C	Crick	SCGN_CpG-23	SCGN	4	0.8
dat_C	Crick	ELOVL2_CpG-82	ELOVL2	3	0.6
dat_W	Watson	KLF14_CpG-65	KLF14	5	1.0
dat_W	Watson	TRIM59_CpG-44	TRIM59	5	1.0
dat_W	Watson	C1orf132_CpG-10	C1orf132	4	0.8
dat_W	Watson	ELOVL2_CpG-67	ELOVL2	4	0.8
dat_W	Watson	PDE4C_CpG-31	PDE4C	3	0.6

Matrix	Strand	CpG	Gene	Folds_selected	Frequency
dat_WC	Watson+Crick collapsed	C1orf132_CpG-10	C1orf132	5	1.0
dat_WC	Watson+Crick collapsed	EDARADD_CpG-2	EDARADD	5	1.0
dat_WC	Watson+Crick collapsed	ELOVL2_CpG-72	ELOVL2	5	1.0
dat_WC	Watson+Crick collapsed	KLF14_CpG-65	KLF14	5	1.0
dat_WC	Watson+Crick collapsed	SCGN_CpG-7	SCGN	5	1.0
dat_WC	Watson+Crick collapsed	PDE4C_CpG-42	PDE4C	3	0.6
dat_WC	Watson+Crick collapsed	TRIM59_CpG-55	TRIM59	3	0.6

Final Model Performance (CV 10×5)

Table 4. Final model performance estimated by repeated internal cross-validation (10-fold × 5-repeat CV) using representative CpGs and natural splines.

Matrix	Strand	n_CpG	n_Splines	MAE	RMSE	R^2
dat_W	Watson	5	2	4.422	5.265	0.885
dat_C	Crick	6	1	4.759	5.803	0.856
dat_WC	Watson+Crick collapsed	7	3	5.085	6.247	0.844

Training Calibration

Table 5. Bias and correlation in full training-set fit.

Matrix	Strand	Mean_bias	SD_error	Cor_age_pred
dat_C	Crick	0	5.498	0.929
dat_W	Watson	0	4.809	0.945
dat_WC	Watson+Crick collapsed	0	5.660	0.924

Model Coefficients

Table 6. Top coefficients of the final Watson-strand Elastic Net model

Term	Estimate	Abs_estimate
C1orf132_CpG-5_ns_3	-6.32490	6.32490
TRIM59_CpG-44	5.65080	5.65080
C1orf132_CpG-5_ns_1	-4.36455	4.36455
C1orf132_CpG-5_ns_2	-2.68013	2.68013
KLF14_CpG-65_ns_2	2.05456	2.05456
PDE4C_CpG-35	2.00196	2.00196

Term	Estimate	Abs_estimate
KLF14_CpG-65_ns_1	1.39271	1.39271
ELOVL2_CpG-67	1.36207	1.36207
KLF14_CpG-65_ns_3	1.32423	1.32423

Comparison algorithms evaluated

Table 7. Comparison of the performance of all six algorithms evaluated: Elastic Net, Ridge regression, LASSO, Random Forest, SVM, and XGBoost.

Matrix	Algorithm	MAE (years)	RMSE (years)	R^2
Watson (dat_W)	**Elastic Net**	**3.94 ± 0.13**	4.98 ± 0.16	0.896 ± 0.008
	LASSO	3.96 ± 0.13	5.01 ± 0.17	0.895 ± 0.008
	SVM	3.96 ± 0.13	5.04 ± 0.19	0.888 ± 0.010
	Ridge	4.01 ± 0.13	5.15 ± 0.19	0.885 ± 0.010
	XGBoost	4.14 ± 0.14	5.32 ± 0.18	0.873 ± 0.009
	Random Forest	4.64 ± 0.13	5.67 ± 0.16	0.871 ± 0.008
Crick (dat_C)	**XGBoost**	**4.56 ± 0.18**	5.89 ± 0.25	0.839 ± 0.017
	Random Forest	4.71 ± 0.18	5.96 ± 0.24	0.838 ± 0.017
	Elastic Net	4.77 ± 0.15	5.82 ± 0.17	0.851 ± 0.009
	LASSO	4.77 ± 0.15	5.82 ± 0.17	0.851 ± 0.009
	SVM	4.82 ± 0.14	5.77 ± 0.15	0.855 ± 0.010
	Ridge	4.96 ± 0.16	6.08 ± 0.17	0.838 ± 0.010
W + C (dat_WC)	**SVM**	**4.42 ± 0.18**	5.69 ± 0.25	0.848 ± 0.015
	Elastic Net	5.24 ± 0.19	6.46 ± 0.23	0.805 ± 0.015
	Ridge	5.24 ± 0.18	6.55 ± 0.25	0.807 ± 0.015
	LASSO	5.29 ± 0.19	6.53 ± 0.25	0.800 ± 0.016
	Random Forest	5.32 ± 0.18	6.40 ± 0.20	0.843 ± 0.013
	XGBoost	5.36 ± 0.21	6.55 ± 0.23	0.809 ± 0.015

Note: These MAEs were calculated on the complete set of candidate CpGs for each matrix (~16-19 CpGs). This explains why the Elastic Net MAE in Watson is 3.94 years in this comparison, whereas the final reduced Watson model with 5 CpGs and natural splines has an MAE of 4.42 years (SM3 Table 4). Both results are consistent: the reduction to 5 CpGs slightly increases the error in exchange for parsimony and interpretability.

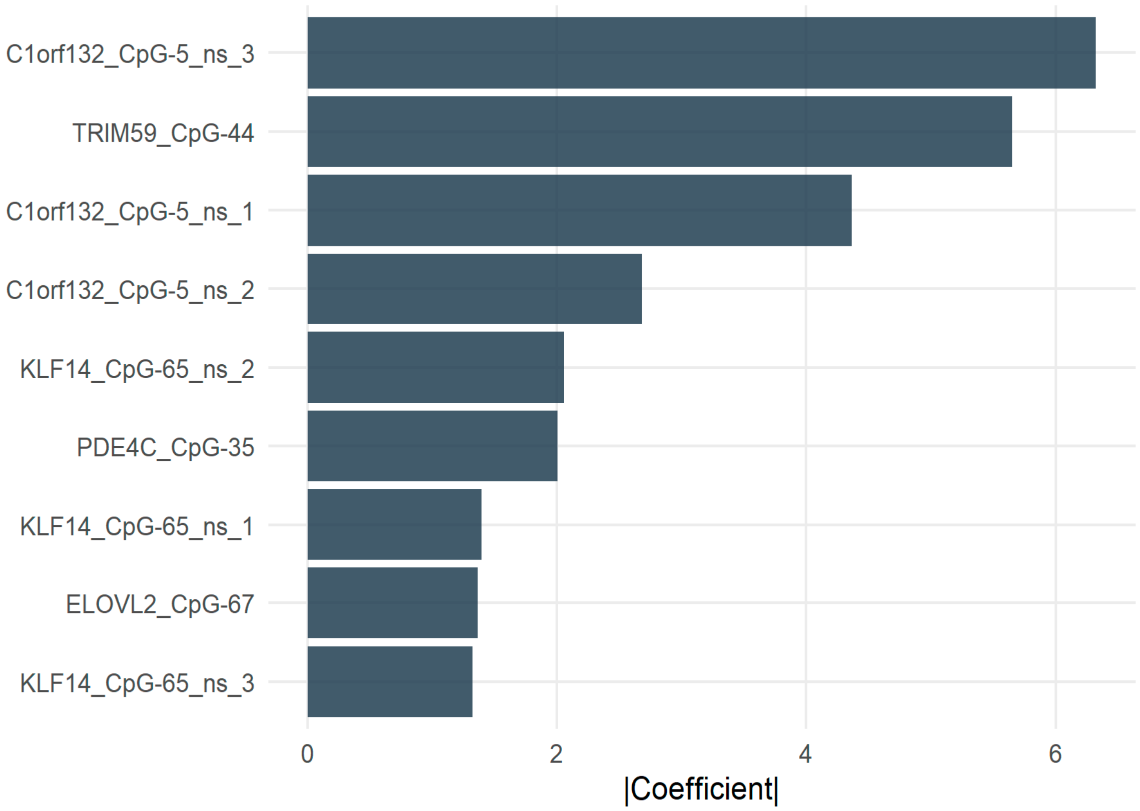

Figure 1. Absolute Elastic Net coefficients for the Watson-strand final model. Spline basis terms are labeled with their CpG of origin.
